# Development of a Doxorubicin Resistance Model in HER2− and HER2+ Breast Cancer to Analyze Potential Therapy Targets and Drug Delivery Methods

**DOI:** 10.3390/cells15151393

**Published:** 2026-07-31

**Authors:** Sara Molenda, Katarzyna Gryska, Igor Piotrowski, Agata Kubicka, Agata Sikorska, Tomasz Deptuch, Hanna Dams-Kozlowska

**Affiliations:** 1 Department of Cancer Immunology, Poznan University of Medical Sciences, 60-806 Poznan, Poland; sara.molenda97@gmail.com (S.M.); agata.sikorska@ump.edu.pl (A.S.); to.deptuch@ump.edu.pl (T.D.); 2 Department of Diagnostics and Cancer Immunology, Greater Poland Cancer Centre, 61-866 Poznan, Poland; 3 Doctoral School, Poznan University of Medical Sciences, 61-701 Poznan, Poland; 4 Department of Medical Physics, Greater Poland Cancer Centre, 61-866 Poznan, Poland; igor.piotrowski@wco.pl; 5 Department of Tumour Pathology and Prophylaxis, Poznan University of Medical Sciences, 61-866 Poznan, Poland; agata.kubicka@wco.pl; 6 Department of Cancer Pathology, Greater Poland Cancer Centre, 61-866 Poznan, Poland

**Keywords:** chemoresistance, breast cancer, HER2, Stat3, siRNA, targeted drug delivery, silk spheres

## Abstract

**Highlights:**

**What are the main findings?**

- The chemotherapy-resistant HER2− (D2F2/Dox) and HER2+ (D2F2E2/Dox) exhibited distinct morphology, proliferation, and migration compared with parental cells and with each other.
- Although chemoresistance in both models was associated with increased Stat3 activation, the mechanisms of chemoresistance acquisition were distinct.
- Stat3 silencing by transfection or using a drug delivery system in D2F2/Dox and D2F2E2/Dox did not restore drug sensitivity in cells.

**What are the implications of the main findings?**

- STAT3 is a promising therapeutic target in HER2-positive and HER2-negative tumors, but effective treatment may require tailored combinatory therapeutic strategies.
- The model of a doxorubicin resistance model in HER2- and HER2+ breast cancer has been established for analyzing potential therapy targets and drug delivery methods.

**Abstract:**

Despite the development of new drugs, chemoresistance constitutes a major obstacle in cancer treatment. To investigate mechanisms of resistance and potential therapeutic targets, we developed doxorubicin-resistant models of HER2− (D2F2/Dox) and HER2+ (D2F2E2/Dox) breast cancer cells. Compared with parental cells, the D2F2/Dox and D2F2E2/Dox differed in morphology, increased migratory potential, elevated levels of the transcription factor signal transducer and activator of transcription 3 (Stat3), and a lower proliferation rate in D2F2E2/Dox. Moreover, D2F2/Dox and D2F2E2/Dox differed in the expression profiles of genes related to cell stemness, apoptosis, and drug efflux. *Stat3* gene silencing in both doxorubicin-resistant cell types reversed the expression profiles of some genes (different in each resistant cell line), and decreased migratory potential was observed only in D2F2 cells. These data indicate that the acquired doxorubicin resistance was associated with Stat3 status; however, HER2− and HER2+ breast cancer cells did not indicate the same mechanism of chemoresistance acquisition. Importantly, Stat3 silencing did not substantially restore doxorubicin sensitivity, suggesting that effective therapy may require simultaneous targeting of multiple pathways. Furthermore, we demonstrated that si*Stat3* therapeutics could be selectively delivered to HER2+ cancer cells using H2.1MS1:MS2KN silk spheres, indicating their potential for targeted drug delivery in vivo.

## 1. Introduction

Breast cancer (BC) is among the most prevalent malignant tumors affecting women and remains the leading cause of cancer-related mortality [1,2]. Based on molecular classification, BC can be divided into four main subtypes: luminal A, luminal B, HER2-positive (HER2+), and triple-negative (TNBC), depending on the expression status of the following factors: estrogen receptor (ER), progesterone receptor (PR), and human epidermal growth factor receptor (HER2) [3]. Luminal A tumors are characterized as ER+, PR+/−, HER2-; luminal B—ER+, PR+/−, HER2+/−; HER2-positive—ER−, PR−, HER2+, and TNBC as ER−, PR−, HER2− [3]. The expression status of hormone receptors, including ER and PR, represents an important determinant of breast cancer biology, prognosis, and therapeutic response [4]. HR-positive and HR-negative tumors exhibit distinct molecular characteristics and differences in sensitivity to systemic therapies [5]. Moreover, extensive crosstalk between hormone receptor signaling and HER2 pathways contributes to breast cancer heterogeneity and may influence treatment response and the development of resistance mechanisms [6]. The present study focuses specifically on HER2-associated mechanisms, while acknowledging that other receptor pathways may also contribute to the complexity of breast cancer biology and therapeutic response.

HER2 is a proto-oncogene that is amplified in approximately 15–25% of breast cancers [7]. It belongs to the epidermal growth factor receptor (EGFR) family of tyrosine kinase receptors, which are located on the cell membrane. HER2 overexpression is associated with poor prognosis, early relapse, enhanced tumor growth, and metastatic potential [8,9,10]. Clinical evidence indicates that HER2-positive tumors display reduced responsiveness to chemotherapy [11]. Compared with patients with HER2-negative breast tumors, the HER2+ breast cancer patients showed a significantly lower response to cyclophosphamide, methotrexate, 5-fluorouracil, prednisone, and epirubicin [12,13,14]. Additionally, numerous in vitro studies have shown that HER2-expressing breast cancer cells are less sensitive to paclitaxel, docetaxel, doxorubicin, 5-fluorouracil, etoposide, and camptothecin [15,16,17,18]. Despite these findings, the mechanisms underlying chemoresistance in HER2-positive breast cancer remain incompletely understood and warrant further investigation.

Chemoresistance in cancer arises from the interplay of multiple biological processes, including enhanced drug efflux, activation of survival signaling pathways, and phenotypic plasticity [19]. Additionally, enrichment of cancer stem-like cells (CSCs) within chemotherapy-resistant cell populations has been observed [20,21]. In breast cancer, CSCs, particularly those with the CD44high/CD24low/ALDH+ phenotype, exhibit increased DNA repair capacity, self-renewal ability, and quiescence, which enables their survival during chemotherapy and predisposes them to relapse [22,23]. Concurrently, ATP-binding cassette (ABC) transporters, such as MDR1 (encoded by *ABCB1*), MRP1 (*ABCC1*), and BCRP (*ABCG2*), reduce intracellular drug accumulation, thereby contributing to resistance to anthracyclines and taxanes [24,25]. Because cells utilize complex regulatory interplay and feedback loops to respond to environmental stimuli, drug pressure on one pathway can activate other pathways, leading to cancer cells acquiring chemoresistance and ultimately causing therapy failure. The hyperactivation of Signal Transducer and Activator of Transcription 3 (STAT3), frequently driven by IL-6/JAK signaling or receptor tyrosine kinases, is one of the signaling pathways that plays a significant role in the acquisition of chemoresistance, contributing to the cell characteristics mentioned above [26].

Constitutive activation of STAT3 is a hallmark of many solid tumors, including breast cancer [27,28,29,30]. Constant STAT3 signaling drives oncogenic programs by upregulating genes associated with cell proliferation (*CCND1*, *c-MYC*), anti-apoptosis (*BCL2*, *MCL1*, *BIRC5*), angiogenesis (*VEGF*), epithelial–mesenchymal transition (EMT) (*TWIST*, *ZEB1/2*), metabolism (*FASN*, *PKM2*, *SLC1A5*), tumor immunosuppression (*IL6*, *TGFb*, *VEGF*, *PD-L1*, *CTLA4*), stemness (*ALDH*, *NANOG*, *SLUG*), and also with chemoresistance [28]. Numerous communications indicated the significant role of STAT3 in chemoresistance in various types of breast cancer and its inhibition as a viable strategy to overcome therapy resistance. In a TNBC model, doxorubicin exposure selectively upregulated STAT3 within the BCSC-enriched (CD44high/CD24low/ALDH+) fraction, highlighting the role of STAT3 in stemness-driven resistance [31]. Cheng et al. reported that a STAT3/Oct-4/c-Myc signaling circuit regulated stemness-mediated doxorubicin resistance in TNBC [32]. Continuous doxorubicin stimulation activated STAT3 and increased Oct-4 and c-Myc levels, enriching CSCs and conferring TNBC cell resistance to the drug. Additionally, therapy with the STAT3 inhibitor WP1066 overcomes resistance to doxorubicin by inhibiting p-STAT3, decreasing Oct-4 and c-Myc levels, and reducing CSC levels. Interestingly, in the same study, in ER-positive, non-metastatic MCF-7 breast cancer cells, doxorubicin resistance was induced by STAT3 activation and upregulation of superoxide dismutase 2 (SOD2) (not observed in TNBC), leading to CSC enrichment [32]. MCF-7-derived tamoxifen-resistant cell line showed increased levels of phosphorylated Src, EGFR(Y845), and STAT3(Y705) [33]. Both a STAT3 inhibitor (stattic) and knockdown of STAT3 expression enhanced sensitivity to tamoxifen in tamoxifen-resistant cells [33]. The highly metastatic MDA-MB-231 TNBC was found to express higher baseline levels of (activated) p-Tyr(705)STAT3, which was further upregulated in doxorubicin-resistant cells [34]. The use of three STAT3 inhibitor types increased breast cancer cell response to the drugs [34]. Additionally, preclinical studies have shown that STAT3 inhibition yields significant therapeutic benefit. The STAT3 inhibitor C188 markedly reduced tumor-initiating cells (CD44+/CD24−/low/ALDH+), mammosphere formation, and delayed recurrence in docetaxel-resistant TNBC xenografts, improving recurrence-free survival nearly fourfold compared with chemotherapy alone [35].

The role of STAT3 in drug resistance in HER2-positive breast cancer cells is more intricate. The activated HER2 (homodimers and heterodimers) interacts with signaling proteins, leading to activation of various signaling pathways, including, but not limited to, the SRC-PTEN-phosphoinositide 3-kinase (PI3K)-AKT pathway, the RAS-RAF-extracellular signal-regulated kinase (ERK) pathway, and the JAK/STAT3 pathway, promoting cell migration, survival, and proliferation [36]. The signaling through HER2 leads to the phosphorylation and activation of STAT3; however, Src, but not Jak kinase, activity is required for HER2-mediated STAT3 activation [37]. Nevertheless, the activation of STAT3 leads to IL-6 secretion, which further stimulates STAT3, generating an HER2/IL6/STAT3 loop [38]. As mentioned above, in TNBC, activated STAT3 plays a crucial role in maintaining stemness. Numerous data indicate that in HER2+ breast cancer, HER2 regulates STAT-mediated induction of EMT and stemness [39]. In HER2-overexpressing breast cancer cells, STAT3 phosphorylation increased the expression of stem cell markers (Oct4, Sox2, and CD44) and tumor sphere formation, whereas inhibition of HER2 and/or STAT3 abolished the BCSC and EMT phenotypes [40]. Additionally, it was demonstrated that the HER2/IL-6/STAT3 signaling axis is involved in tumorigenesis [41]. Within this loop, STAT3 is required for anchorage-independent growth and metastasis, but not for mammary tumor development [42]. Moreover, activated STAT3 is important for radio(chemo)resistance in HER2+ breast cancer [38]. The HER2-STAT3-survivin axis is involved in radiotherapy resistance of HER2+ breast cancer cells [43]. Hawthorne et al. reported that in breast cancer, HER2 via Src activates STAT3, which upregulates p21Cip1 transcription, leading to Taxol resistance in cancer cells [44]. The drug sensitivity was restored by Src and STAT3 inhibitors in resistant HER2-overexpressing breast cancer cells [44].

Besides the role in the radio(chemo)resistance in HER2+ breast cancer, STAT3 is also a significant factor in resistance development after targeted therapies [45]. Although historically HER2-positive breast cancer was associated with poor prognosis, the introduction of HER2-targeted therapies, such as HER2 antibodies and derivatives, tyrosine kinase inhibitors, and antibody–drug conjugates, significantly improved disease outcome. Unfortunately, after some time of treatment, the disease can progress due to the acquisition of therapy resistance [46]. Among various factors contributing to resistance to HER2-targeted therapy, STAT3 is considered. The resistance to trastuzumab (an anti-HER2 antibody) has been associated with a STAT3-dependent positive feedback loop involving IL-6, EGF, and fibronectin, which sustains STAT3 activation and HER2 signaling [47]. The persistently activated STAT3 leads to increased expression of MUC1 and MUC4, with MUC1 sustaining HER2 signaling and MUC4 physically blocking trastuzumab from binding to HER2. Inhibition of STAT3 (using S3I-201) can re-sensitize resistant cells by blocking this feedback loop, leading to cell death [47]. Furthermore, non-structural maintenance of the chromosome condensin 1 complex subunit G (NCAPG) overexpression reduces cell sensitivity to trastuzumab by activating Src protein kinase and enhancing STAT3 nuclear translocation, promoting cell proliferation and survival [48]. The resistance to trastuzumab-emtansine (T-DM1, an antibody–drug conjugate) in metastatic breast cancer was attributed to STAT3 activation mediated by leukemia inhibitory factor receptor (LIFR) overexpression. Conversely, STAT3 inhibition sensitized resistant cells to T-DM1, both in vitro and in vivo [49].

Given its central role in tumor growth, survival, angiogenesis, metastasis, and therapy resistance across multiple BC subtypes, STAT3 represents an attractive therapeutic target in breast cancer. Targeting STAT3 is particularly important for overcoming therapy resistance [45,50]. Although numerous studies implicate STAT3 in the development of treatment resistance, the precise mechanisms by which STAT3 contributes to this process remain incompletely defined. Further elucidation of these mechanisms is therefore critical to advance targeted therapeutic approaches. Several strategies have been developed to inhibit STAT3 activity, including (i) small-molecule inhibitors that disrupt phosphorylation, dimerization, or DNA binding, (ii) RNA interference approaches that downregulate *STAT3* expression, and (iii) nanoparticle-based delivery systems capable of co-delivering *STAT3* siRNA with chemotherapeutic agents, thereby enhancing efficacy and minimizing off-target effects [28,51]. Several factors can contribute to combat STAT3-dependent therapy resistance, including the breast cancer phenotype (HER2+/-, hormone receptor status), the mechanism of therapy resistance acquisition, the level of STAT3 activation, and the method of STAT3 inhibition.

In this study, we developed models of doxorubicin-resistant murine breast cancer cells with and without human HER2 overexpression. We examined and compared native and chemotherapy-resistant cells for proliferation, migration, and doxorubicin sensitivity. Additionally, the expression of genes associated with chemoresistance and tumor progression was analyzed in relation to Stat3 levels and phosphorylation. Next, the therapeutic effect of siRNA targeting *Stat3* (si*Stat3*) was studied to assess the impact of Stat3 on cellular chemoresistance. Finally, we applied the previously developed silk-based siRNA delivery platform (H2.1MS1/MS2KN nanospheres) to examine its potential for targeted delivery and the silencing effect of si*Stat3* in HER2-overexpressing breast cancer cells. The results indicate that chemoresistance in both HER2-negative and HER2-positive cells was associated with *Stat3* overactivation; however, each cell model appears to exhibit distinct Stat3 phosphorylation patterns, gene expression changes, and proliferation/migration behaviors. Notably, a drug delivery system based on spider silk functionalized with a HER2-targeting ligand effectively delivered siRNA therapeutics to HER2-positive breast cancer cells, representing a promising strategy for combination therapy in this cell type.

## 2. Materials and Methods

### 2.1. Cell Culture

A murine D2F2 and D2F2E2 breast cancer cell lines were a kind gift from Prof. Constantin Baxevanis (Cancer Immunology and Immunotherapy Center, Saint Savas Cancer Hospital, Athens, Greece) and were cultured in DMEM medium supplemented with 10% fetal bovine serum (FBS) and 80 µg/mL gentamycin [52]. The cells were grown at 37 °C in an incubator containing 5% CO_2_. D2F2 cells were modified previously to overexpress the human *ERBB2/neu* gene encoding HER2 to obtain D2F2E2 variant [53]. D2F2E2 cells were routinely cultured in the presence of G418 (200 µg/mL) to maintain stable overexpression of *ERBB2/neu*.

### 2.2. Generation of Chemotherapy-Resistant Breast Cancer Cells

To obtain cells exhibiting chemoresistance to doxorubicin (Dox), the D2F2 and D2F2E2 cells were cultured at gradually increased concentrations of this chemotherapeutic. First, D2F2 and D2F2E2 cells were treated with doxorubicin (Adriamycin, Pfizer Inc., New York, NY, USA) at a concentration of 10 nM. After the cells reached a confluence of 90%, they were passaged and exposed to a higher Dox concentration. Finally, after 6 months of stepwise adaptation to increasing concentrations of doxorubicin, reaching a final maintenance concentration of 1280 nM, stable doxorubicin-resistant cell lines were established and designated D2F2/Dox and D2F2E2/Dox, respectively. 1280 nM Doxorubin is equivalent to a drug concentration of 0.696 μg/mL.

### 2.3. Determination of Half-Maximal Inhibitory Concentration (IC50) of Doxorubicin

To determine the IC50 value of the doxorubicin, the D2F2, D2F2/Dox, D2F2E2 and D2F2E2/Dox cells were seeded at a quantity 2 × 10^4^ per well on a 96-well plate in triplicate. The following day, cells were treated with Dox at a range of concentrations and then incubated for 48 h. Next, the cell’s vitality was assessed by adding 1 mg/mL of 3-(4,5-dimethylthiazol-2-yl)-2,5-diphenyltetrazolium bromide (MTT) (VWR, Radnor, PA, USA), followed by incubation at 37 °C for 4 h. After removing the medium, the formazan crystals were dissolved in Dimethyl Sulfoxide (DMSO) (Chempur, Piekary Śląskie, Poland). The absorbance of the samples was measured using a Victor X2 Multilabel Microplate Reader (PerkinElmer, Waltham, MA, USA) at a wavelength of 560 nm. The experiment was repeated 3 times. The IC50 value was determined by plotting the percentage of cell viability against the logarithm of doxorubicin concentrations and fitting a nonlinear regression curve using GraphPad Prism 9 (GraphPad Software, San Diego, CA, USA).

### 2.4. Analysis of Gene Expression

A total of 2 × 10^5^ cells of D2F2, D2F2/Dox, D2F2E2, and D2F2E2/Dox were seeded per well in a 12-well plate. After 48 h, RNA was isolated using Fenozol (A&A Biotechnology, Gdansk, Poland) according to the manufacturer’s protocol. Subsequently, 1 µg of RNA was used to perform reverse transcription with the iScript Reverse Transcription Supermix (Bio-Rad, Hercules, CA, USA) reagent. cDNA was then subjected to gene expression analysis using Maxima SYBR Green/ROX qPCR Master Mix (ThermoFisher, Waltham, MA, USA) according to the vendor’s protocol. The relative gene expression levels were calculated using the comparative Ct method (ΔCt), with GAPDH serving as the reference gene. For the analysis of gene expression changes following *Stat3* silencing, relative gene expression was calculated using the 2^−ΔΔCt^ method, with si*Luc*-transfected cells as the calibrator, set to 1. Table 1 lists the primers used for gene expression analysis.

### 2.5. The Western Blot Assay

To obtain a protein sample, 1 × 10^6^ of the D2F2, D2F2/Dox, D2F2E2, or D2F2E2/Dox cells were seeded on a 60 mm diameter plate. After 24 h, protein was isolated using RIPA supplemented with cOmpletec proteinase inhibitor cocktail (Roche, Basel, Switzerland) and PhosSTOP phosphatase inhibitor cocktail (Roche, Basel, Switzerland). Cells were incubated with 50 µL of RIPA buffer for 15 min on ice. Subsequently, the lysates were centrifuged at 14,000 rpm for 10 min at 4 °C, and the supernatant was collected. Afterwards, the protein concentration was measured using the Pierce BCA Protein Assay Kit (ThermoFisher, Waltham, MA, USA) according to the vendor’s instructions. A total of 40 µg of protein was loaded onto a 10% polyacrylamide gel (SDS-PAGE) and separated by electrophoresis. The protein was then transferred onto a nitrocellulose membrane (Bio-Rad, Hercules, CA, USA) using the Trans-Blot Turbo System (Bio-Rad, Hercules, CA, USA). The membrane was blocked with non-fat dry milk, followed by overnight incubation with the primary antibodies at a 1:1000 dilution, i.e., anti-β-actin (Santa Cruz Biotechnology, Dallas, TX, USA, (clone C4), sc-47778), anti-Stat3 (Santa Cruz Biotechnology, Dallas, TX, USA, Stat3 Antibody (clone F-2), sc-8019), anti-p-Stat3 (Tyr705) (AbClonal, Woburn, MA, USA, ARC50831), or anti-p-Stat3 (Ser727) (AbClonal, Woburn, MA, USA, ARC0150). Subsequently, the appropriate HRP-conjugated secondary antibodies (anti-mouse IgG (Sigma, St. Louis, MO, USA) or anti-rabbit IgG (Sigma, St. Louis, MO, USA), were applied at a 1:3000 dilution. The signal was excited with ECL SuperSignal reagent (ThermoFisher, Waltham, MA, USA) and then detected using a G-Box imaging system (Syngene, Frederick, MD, USA).

### 2.6. Flow Cytometry Analysis

A total of 1 × 10^5^ D2F2, D2F2/Dox, D2F2E2, and D2F2E2/Dox cells were incubated with fluorochrome-conjugated antibodies for surface marker detection. Human HER2 expression was analyzed using Anti-HER2 antibody (Pfizer, Andover, MA, USA), which was first conjugated with the FITC fluorophore (Sigma, St. Louis, MO, USA) according to the supplier’s protocol. The Cd44 and Pd-l1 expression were detected using APC-conjugated anti-Cd44 (clone IM7, Immunotools, Friesoythe, Germany) and Alexa Fluor 647-conjugated anti-Pd-l1 (clone MIH5, BD Biosciences, San Diego, CA, USA) antibodies, respectively. Cells were incubated with the antibodies for 30 min at 4 °C in the dark according to the manufacturer’s recommendations. Following incubation, the cells were washed with PBS to remove unbound antibodies and resuspended in PBS for analysis.

Samples were analyzed using a flow cytometer, a DxFLEX (Beckman Coulter, Brea, CA, USA) or Navios (Beckman Coulter, Brea, CA, USA), and the data were processed using FlowJo software (version 10.10.0, BD Biosciences, Ashland, OR, USA).

### 2.7. The Cell Proliferation Assay

The D2F2, D2F2/Dox, D2F2E2, and D2F2E2/Dox cells were seeded at a density of 5 × 10^3^ cells per well in a 96-well plate. Next, cell proliferation was monitored in real-time using the IncuCyte system (Sartorius, Göttingen, Germany). Images were captured every 4 h, and the cell confluence was subsequently quantified using the IncuCyte analysis software (version 2022BRev2, Sartorius, Göttingen, Germany). The experiment was conducted three times in triplicate.

### 2.8. The Wound Healing Assay

A total of 4 × 10^4^ D2F2, D2F2/Dox, D2F2E2, D2F2E2/Dox cells and D2F2/Dox, D2F2E2/Dox, following si*Stat3* or si*Luc* treatment, were seeded per well in a 96-well plate. The next day, cell proliferation was inhibited by incubating the cells for 3 h in culture medium containing mitomycin C (Zydus Lifesciences Ltd., Ahmedabad, Gujarat, India) at a final concentration of 25 µg/mL. Following the treatment, a scratch was introduced using the Woundmaker tool (Sartorius, Göttingen, Germany). The plate was then placed in the IncuCyte system, which captured images every 3 h. The wound healing rate was quantified using the IncuCyte software with the wound healing analysis module. The experiment was performed three times in triplicate.

### 2.9. The Stat3 Silencing Using Lipofectamine Transfection Reagent

To analyze the impact of *Stat3* silencing on the expression of other genes, D2F2/Dox and D2F2E2/Dox cells were seeded at a quantity of 2 × 10^5^ cells per well on a 12-well plate. The next day, the cells were transfected with *Stat3* siRNA (si*Stat3*) or *Luc* siRNA (si*Luc*) using Lipofectamine RNAiMAX (ThermoFisher, Waltham, MA, USA) according to the manufacturer’s protocol. Briefly, for each well, 2 µL siRNA (10 µM; 20 pmol) was diluted in 100 µL Opti-MEM medium. In a separate tube, 6 µL Lipofectamine RNAiMAX reagent was diluted in 100 µL Opti-MEM medium. The diluted siRNA and Lipofectamine RNAiMAX were incubated separately for 5 min at room temperature, then combined gently and incubated for an additional 5 min to allow siRNA–lipid complex formation. A total of 100 µL siRNA–lipid complex was added dropwise to each well containing cells and complete growth medium. After 48 h, RNA or protein was isolated, and the material was analyzed as described in points 2.4 and 2.5. Additionally, the proliferation and wound healing assays were conducted on si*Stat3*- and si*Luc*-treated cells, following the procedures described in Section 2.6 and Section 2.7.

### 2.10. Analysis of Doxorubicin Effect on Cytotoxicity of Chemotherapy-Resistant Cells with Silenced Stat3 Gene Expression

Cells D2F2/Dox and D2F2E2/Dox, following si*Stat3* or si*Luc* (control) treatment, were seeded into 96-well plates at a density of 10,000 cells per well. The following day, doxorubicin was added at a concentration of 64 µg/mL. Next, the cells were imaged every 2 h for 48 h using the IncuCyte system. The picture-based real-time cell confluency measurements were performed and then analyzed using the IncuCyte software. The experiment was performed three times in triplicate.

### 2.11. Production and Purification of Bioengineered Spider Silks

The production of H2.1MS1 and MS2KN proteins at a laboratory scale was carried out using a Bioflo 415 fermentor (New Brunswick Scientific, Edison, NJ, USA). The H2.1MS1 silk protein was purified by using the thermal method (80:20), as previously described [31]. Briefly, the bacterial pellet was resuspended in a lysis buffer containing 100 mM NaCl, 20 mM HEPES (4-(2-hydroxyethyl)-1-piperazineethanesulfonic acid), pH 7.5, and Pierce Protease Inhibitor, EDTA-free (ThermoFisher, Waltham, MA, USA). To lyse the bacteria, lysozyme (Sigma, St. Louis, MO, USA) was added at a concentration of 0.2 mg/mL, and the mixture was then incubated at 4 °C with agitation for 30 min. Sonication was also performed to disrupt the bacteria and their structures further. DNA was degraded by treatment with DNase I (Sigma, St. Louis, MO, USA) at a concentration of 0.1 mg/mL, in the presence of 3 mM MgCl_2_ (Sigma, St. Louis, MO, USA). Bacterial proteins were then denatured by heating the solution at 80 °C for 10 min and subsequently removed by centrifugation at 21,000× *g* for 30 min at 4 °C. The procedure was repeated for an additional 15 min incubation at 80 °C, followed by centrifugation under the same conditions. The protein was then precipitated overnight at 4 °C using 20% ammonium sulfate (VWR, West Chester, PA, USA). The following day, the silk protein was collected by centrifugation (30 min, 7000× *g*, at RT), and the pellet was rinsed with 20% ammonium sulfate. Next, the precipitated silk was dissolved in 6 M guanidine thiocyanate (Sigma, St. Louis, MO, USA) and dialyzed using cellulose dialysis tubing (14,000 Da cut-off) (Sigma, St. Louis, MO, USA) against a 10 mM TRIS-HCl buffer, pH 7.5.

MS2KN was purified using the same method with certain modifications. First, during DNase I treatment, the concentration of MgCl_2_ was increased up to 70 mM. Additionally, the first incubation at 80 °C was extended to 15 min, while the second was prolonged to 30 min. Otherwise, the standard protocol was followed.

### 2.12. Production of Silk Spheres Loaded with siRNA

To load the oligonucleotide therapeutics into spheres, 50 µL of si*Stat3* or si*Luc* (20 µM) was mixed with 10 µL of MS2KN (1 mg/mL) and incubated for 5 min. The siRNA-silk protein mixture was then combined with 40 µL of H2.1MS1 silk (1 mg/mL). The resulting complexes were vigorously mixed with 1 mL of 2 M potassium phosphate, pH 8.0. The resulting nanoparticles were incubated overnight at RT. The next day, the spheres were dialyzed against 2 L of ultrapure water using membranes with a 14,000 Da molecular weight cut-off. The water was replaced seven times during the dialysis process. After dialysis, the spheres were centrifuged at 21,000× *g* for 1 h at room temperature and resuspended in PBS. The efficiency of siRNA loading into silk spheres was determined based on previously established protocol [54].

### 2.13. Silencing of Stat3 Using Silk Spheres

D2F2/Dox and D2F2E2/Dox cells were seeded into a 12-well plate at a density of 2 × 10^5^ cells per well. The following day, cells were treated with 250 nM si*Luc* or si*Stat3* loaded into H2.1MS1:MS2KN spheres. After 48 h, RNA was isolated from the cells, and gene expression analysis was performed as previously described in Section 2.4.

### 2.14. Statistics

To assess significant differences between D2F2 vs. D2F2/Dox, D2F2E2 vs. D2F2E2/Dox, cells treated with si*Luc* vs. si*Stat3* groups, a *t*-test was applied to samples exhibiting a normal distribution. The Mann–Whitney test was used to determine statistical significance for data not meeting the normality assumption. For experiments involving two independent variables, a two-way ANOVA was applied to evaluate the effects of both factors and their interaction. Results were considered statistically significant at the following *p*-values: **** *p* < 0.0001, *** *p* < 0.001, ** *p* < 0.01, and * *p* < 0.05. Data are presented as mean ± standard error of the mean (SEM). Statistical analyses were performed using GraphPad Prism 8 software.

## 3. Results and Discussion

### 3.1. Generation of Doxorubicin-Resistant D2F2 and D2F2E2 Breast Cancer Cells Derivatives

**Figure 1:**
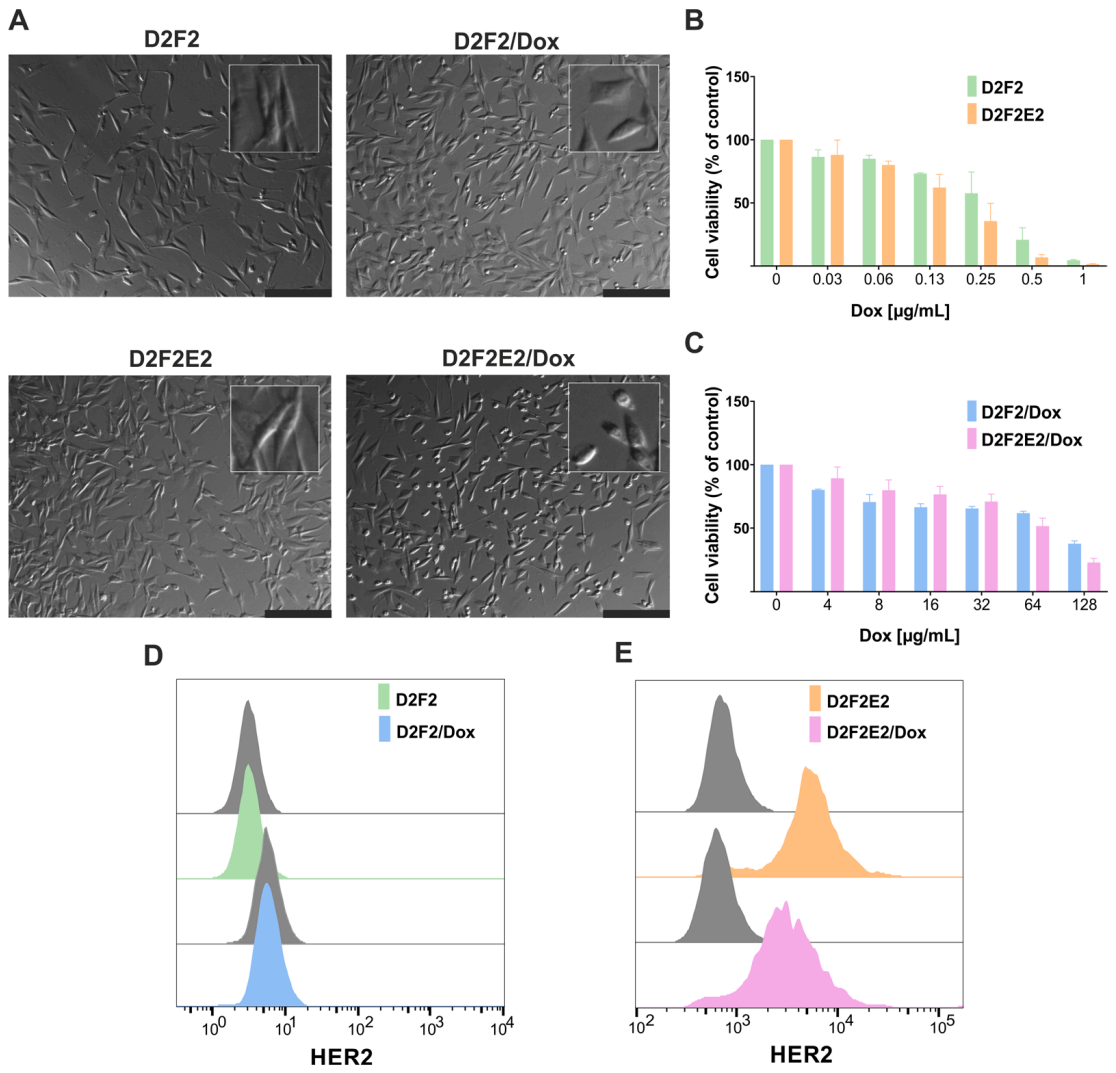
Generation of chemotherapy-resistant HER2− and HER2+ breast cancer cells. (**A**) morphology of chemotherapy-sensitive (**left**) and chemotherapy-resistant (**right**) cell lines without (D2F2, D2F2/Dox) and with (D2F2E2, D2F2E2/Dox) HER2 overexpression. Images were acquired using a Leica DMI3000 B microscope at 10× magnification, scale bar 200 μm. Insets show higher digital magnification of representative cells. (**B**,**C**) Doxorubicin cytotoxicity assay. Chemotherapy-sensitive (**B**) and chemotherapy-resistant (**C**) cells were incubated with varying concentrations of doxorubicin for 48 h, and cell viability was subsequently assessed using the MTT assay. Each experiment was performed three times in triplicate (mean ± SEM). Data were analyzed using GraphPad Prism. (**D**,**E**) Representative flow cytometry histograms analyzing the surface presence of HER2 in (**D**) D2F2, D2F2/Dox, (**E**) D2F2E2, and D2F2E2/Dox cells. HER2 was detected using FITC-conjugated anti-HER2 antibody. Flow cytometry data were analyzed using FlowJo software (version 10.10.0; BD Biosciences). Results are representative of a single flow cytometry experiment.

D2F2 and D2F2E2 are murine mammary carcinoma cell lines. D2F2E2 cells were generated by introducing human *ERBB2/neu* overexpression (HER2) into the parental D2F2 cell line [53]. Doxorubicin-resistant derivatives of D2F2 and D2F2E2 breast cancer cells were generated by stepwise adaptation to escalating doses of doxorubicin. The chemotherapy-resistant derivatives exhibited altered morphology compared to the parental cells (Figure 1A). The chemotherapy-sensitive lines D2F2 and D2F2E2 displayed a predominantly homogeneous population of polygonal-shaped, but slightly elongated, cells with well-defined cell borders, strong substrate adhesion, minimal intercellular spaces, and growth in closely apposed, confluent sheets. This cohesive arrangement is characteristic of epithelial-like parental cultures, which typically retain organized actin cytoskeletal structures and robust cell–cell junctions, features associated with sensitivity to chemotherapeutic agents [55]. In contrast, the chemotherapy-resistant lines, named D2F2/Dox and D2F2E2/Dox, exhibited a markedly more heterogeneous morphology. Alongside elongated cells, a substantial fraction appeared more rounded and less flattened, with irregular colony edges, enlarged intercellular gaps, and, among the D2F2E2/Dox group, a notable proportion of cells were rounded and appeared to be in a pre-detachment state. These observations align with the known manifestations of cytoskeletal remodeling and reduced cell–cell adhesion—key hallmarks of chemotherapy-resistant cell phenotype [55]. Moreover, chemotherapy-resistant cancer cells often exhibit traits associated with cancer stem-like cells, including diminished adhesion, which has been linked to augmented survival under chemotherapeutic stress [56]. Thus, the morphological plasticity observed in D2F2 and D2F2E2 drug-resistant cells likely reflects an adaptive response to chemotherapeutic pressure.

The parental D2F2 and DF2E2 cells were drug sensitive (Figure 1B) exhibiting for doxorubicin IC50 value of 0.31 ± 0.12 µg/mL and 0.18 ± 0.07 µg/mL, respectively (Figure 1D). Following the development of chemoresistance, the D2F2/Dox and D2F2E2/Dox breast cancer cells demonstrated for doxorubicin IC50 values of 90.09 ± 8.63 and 67.45 ± 19.49, respectively (Figure 1C). Based on the obtained results, the doxorubicin-resistant cells have been successfully generated. Moreover, HER2-overexpressing cells exhibited lower IC50 values compared to their counterparts without HER2-overexpression; however, the difference was not significant. Sensitivity to anthracyclines (e.g., doxorubicin) was observed to be elevated in HER2-overexpressing cancer cells [57]. However, in such cases, an increased drug sensitivity was due to co-amplification of the topoisomerase-2 gene that constitutes the target of the drug [58]. Because HER2 amplification was generated by genetic engineering in our model, co-amplification of the topoisomerase-2 gene is unlikely to occur.

To confirm HER2 status in both chemosensitive and chemoresistant cell lines, flow cytometric analysis was performed using a FITC-conjugated anti-human HER2 antibody. This analysis confirmed the absence of human HER2 protein in both D2F2 and its doxorubicin-resistant counterpart, D2F2/Dox (Figure 1D). In contrast, D2F2E2 cells and D2F2E2/Dox cells were positive for HER2 expression (Figure 1E). A uniform rightward shift in the fluorescence intensity peak was observed, suggesting that HER2 expression is present throughout the cell population, albeit at variable levels among individual cells.

### 3.2. Proliferation and Migration of Chemotherapy-Resistant Breast Cancer Cells Differ from Doxorubicin-Sensitive Counterparts

**Figure 2:**
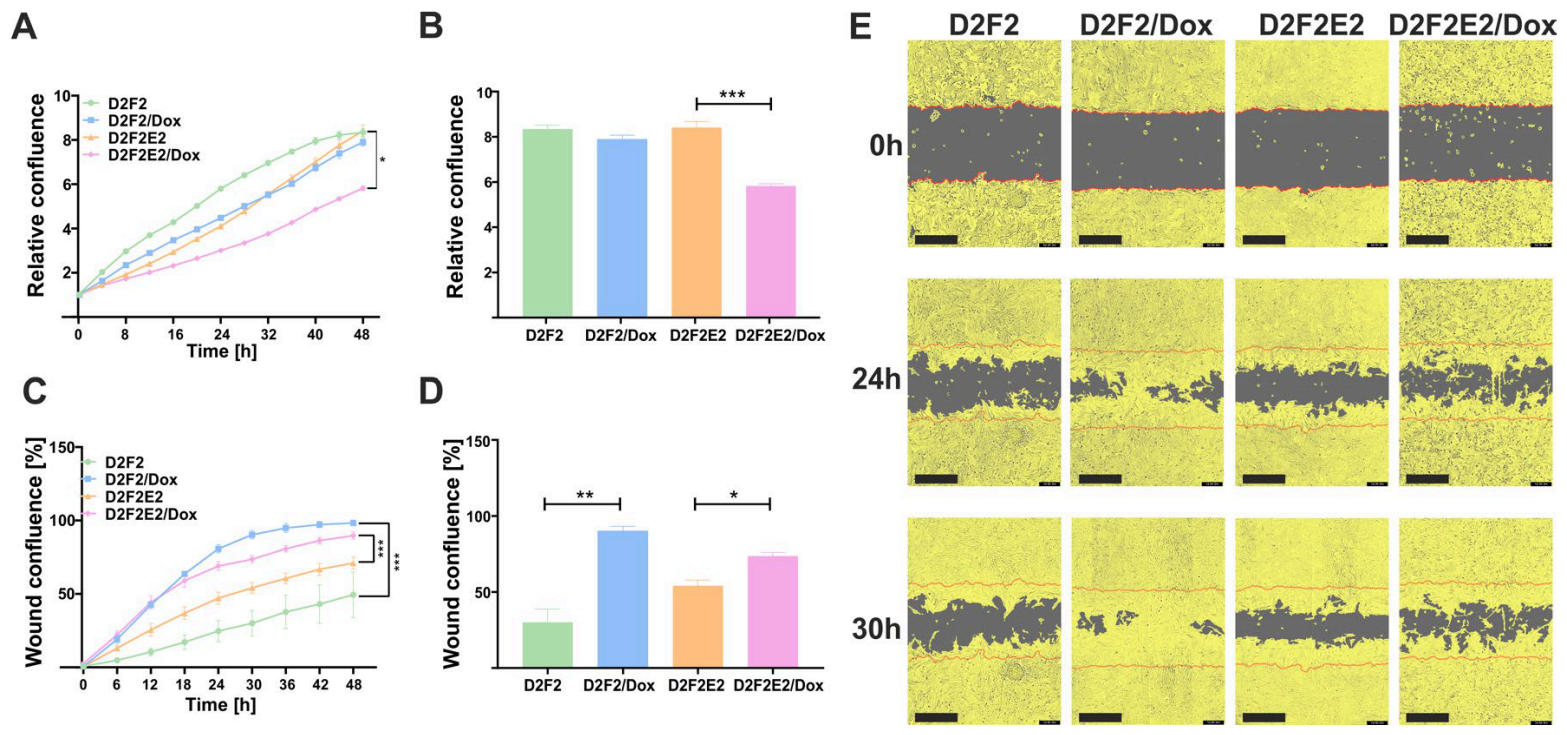
Proliferation and migration of chemotherapy-sensitive and -resistant breast cancer cells. (**A**,**B**) Cell proliferation was assessed by measuring real-time cell confluence using the IncuCyte system. (**A**) Cell proliferation over time. (**B**) Cell confluence after 48 h. (**C**,**D**) Cell migration was evaluated by measuring the wound healing rate in real-time using the IncuCyte system. (**C**) Wound confluence over time. (**D**) Wound Confluence after 30 h. (**E**) Representative images of the wound healing assay at 0, 24, and 30 h time points; scale bar 400 μm (bottom left). Each experiment was performed three times in triplicate (mean ± SEM). Data were analyzed using GraphPad Prism. * Indicates *p* ≤ 0.05, ** *p* ≤ 0.01, and *** *p* < 0.001.

The chemotherapy-resistant cells were characterized for phenotypic and functional features, focusing on proliferation, migration capacity, and gene expression profiles, and compared with parental cells. Doxorubicin-resistant HER2-overexpressing breast cancer cells proliferated significantly slower compared with other cell variants (Figure 2A,B). Reduced proliferation of chemotherapy-resistant cancer cells is a frequently observed phenomenon, associated with a shift toward a more stem-like phenotype [59,60]. The wound healing assay showed that doxorubicin-resistant breast cancer cells exhibited a significantly enhanced migratory potential compared with their drug-sensitive counterparts, regardless of HER2 status (Figure 2C,D). Chemotherapy-resistant cancer cells often exhibit an elevated migratory capacity, which promotes tumor progression and metastasis [61]. In ovarian carcinoma, mesenchymal-like chemotherapy-resistant cells migrated rapidly due to accelerated focal adhesion turnover, mediated by proteins such as FAK, paxillin, vinculin, and talin [62]. Polyploid giant cancer cells (PGCCs), which are multinucleated, chemotherapy-resistant cancer cells found in heterogeneous solid tumors, including breast cancer, have a unique polarized vimentin network that drives their elevated migration [63]. The mentioned adaptations highlight increased migration as a hallmark of chemotherapy-resistant cells and indicate a potential therapeutic vulnerability of chemotherapy-resistant cancer cells [63,64,65].

### 3.3. Chemotherapy-Resistant Breast Cancer Cells Have Upregulated Transcription Factor Stat3

**Figure 3:**
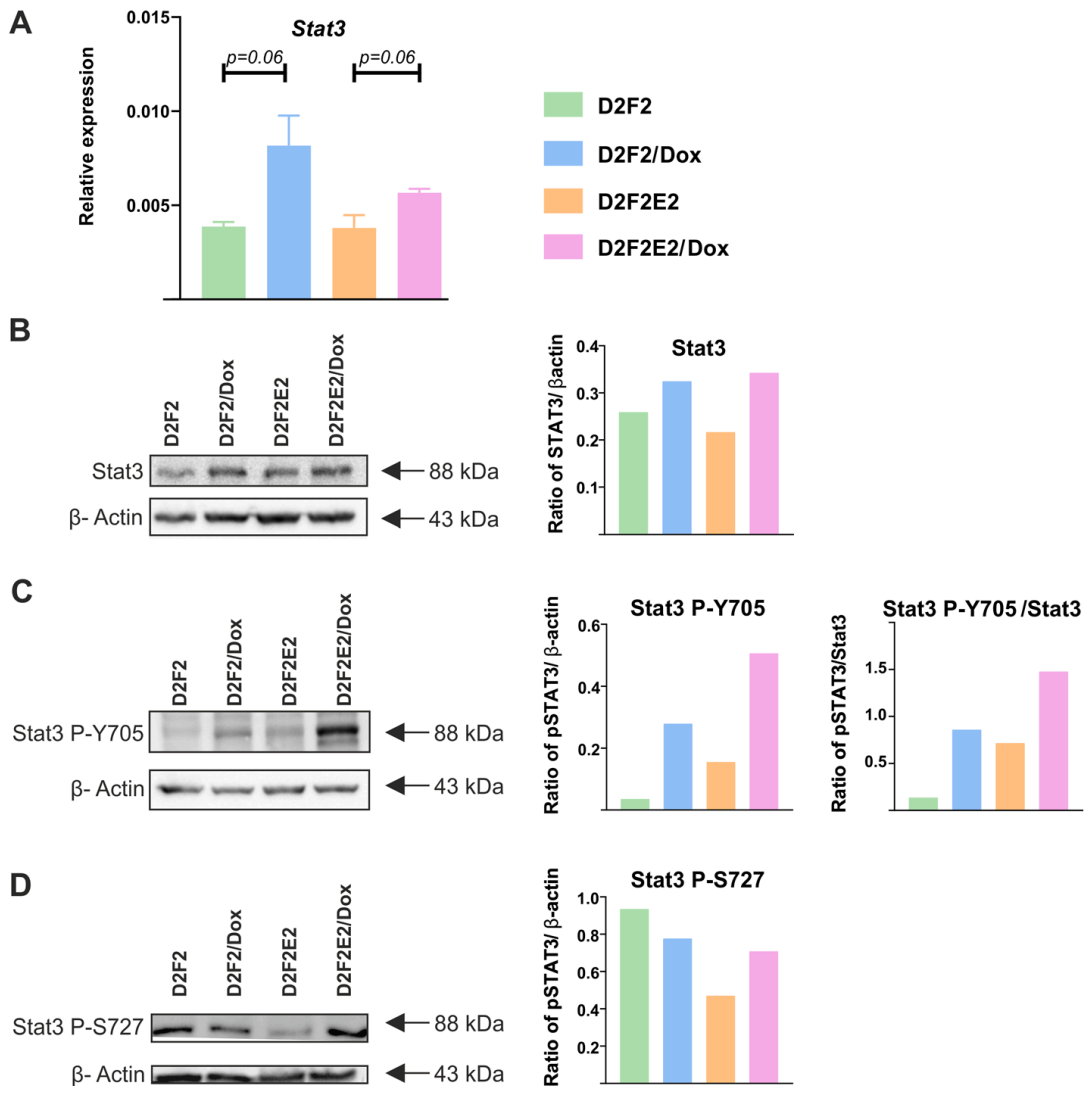
Stat3 mRNA and protein level in chemotherapy-sensitive and -resistant breast cancer cells. (**A**) RNA was isolated from chemotherapy-sensitive and -resistant cancer cell lines, and gene expression was analyzed by qPCR. Gene expression level was normalized to GAPDH. The experiment was performed three times in duplicate. Data were analyzed using GraphPad Prism. (**B**–**D**) Protein was extracted from chemotherapy-sensitive and -resistant cell lines in the presence of protease and phosphatase inhibitors, and Western blot analysis was performed to compare: (**B**) total Stat3 protein, (**C**) phosphorylated Stat3 at Y705 (Stat3 P-Y705), (**D**) phosphorylated Stat3 at S727 (Stat3 P-S727). Changes in protein expression levels were determined by densitometric analysis and normalized to β-actin as a loading control. Additionally phosphorylated STAT3 levels were calculated relative to the corresponding total STAT3 protein levels (Stat3 P-Y705/Stat3). The data from one experiment for each Western Blot is shown.

The analysis of *Stat3* expression indicated that drug-resistant breast cancer cells upregulated the *Stat3* expression (*p* = 0.06) regardless to the HER2-status (Figure 3A). Moreover, the level of basal expression of *Stat3* in drug-sensitive cancer cells was similar for both cancer types (Figure 3A). It was also examined the level of both non-phosphorylated and phosphorylated forms of the Stat3 protein (Figure 3B–D). The analysis indicated a trend in a slight increase in total Stat3 protein levels for both D2F2/Dox and D2F2E2/Dox cancer cells compared with drug-sensitive counterparts (Figure 3B). However, the phosphorylated Stat3 (Stat3 P-Y705) was considerably elevated in doxorubicin-resistant cells (Figure 3C). It is worth to notice, that the level of Stat3 P-Y705 was also higher in HER2-overexpressing D2F2E2 cells than in D2F2 variant (Figure 3C). Additionally, the drug-resistant HER2-overexpressing cells exhibited increased levels of the alternatively activated form of Stat3 protein (Stat3 P-S727) relative to the Dox-sensitive cells (Figure 3D). STAT3 activation is primarily mediated through phosphorylation at tyrosine 705 (Y705), typically catalyzed by receptor-associated kinases such as JAKs upon cytokine (e.g., IL-6) or growth factor (e.g., EGF) signaling [64]. P-Y705 modification drives STAT3 dimerization, nuclear translocation, and DNA binding to regulate transcription of genes involved in proliferation and survival [66]. Additionally, phosphorylation at serine 727 (S727)—triggered by MAPKs, mTOR, or other kinases—amplifies STAT3’s transcriptional potency and facilitates its mitochondrial functions, such as modulation of cellular respiration and reactive oxygen species production [64]. Notably, P-S727 can modulate the duration of STAT3 activity by promoting the dephosphorylation of Y705 via nuclear phosphatases, such as TC45 [67]. Increased activation and expression of STAT3 were observed in chemotherapy-resistant cancer cells, including breast cancer [45,68,69]. Moreover, Chung et al. demonstrated that HER2 can promote STAT3 phosphorylation [70], findings that are consistent with our study, in which chemotherapy-resistant cancer cells with HER2 overexpression exhibited higher levels of phosphorylated Stat3 than their counterparts lacking HER2 overexpression. Thus, considering both the increased expression of Stat3 and the increased abundance of phosphorylated Stat3 protein in drug-resistant cancer cells, Stat3 may be associated with the acquisition of chemotherapy resistance and represent an attractive therapeutic target.

### 3.4. Analysis of the Expression of Genes Related to the Stat3 Transcription Factor

**Figure 4:**
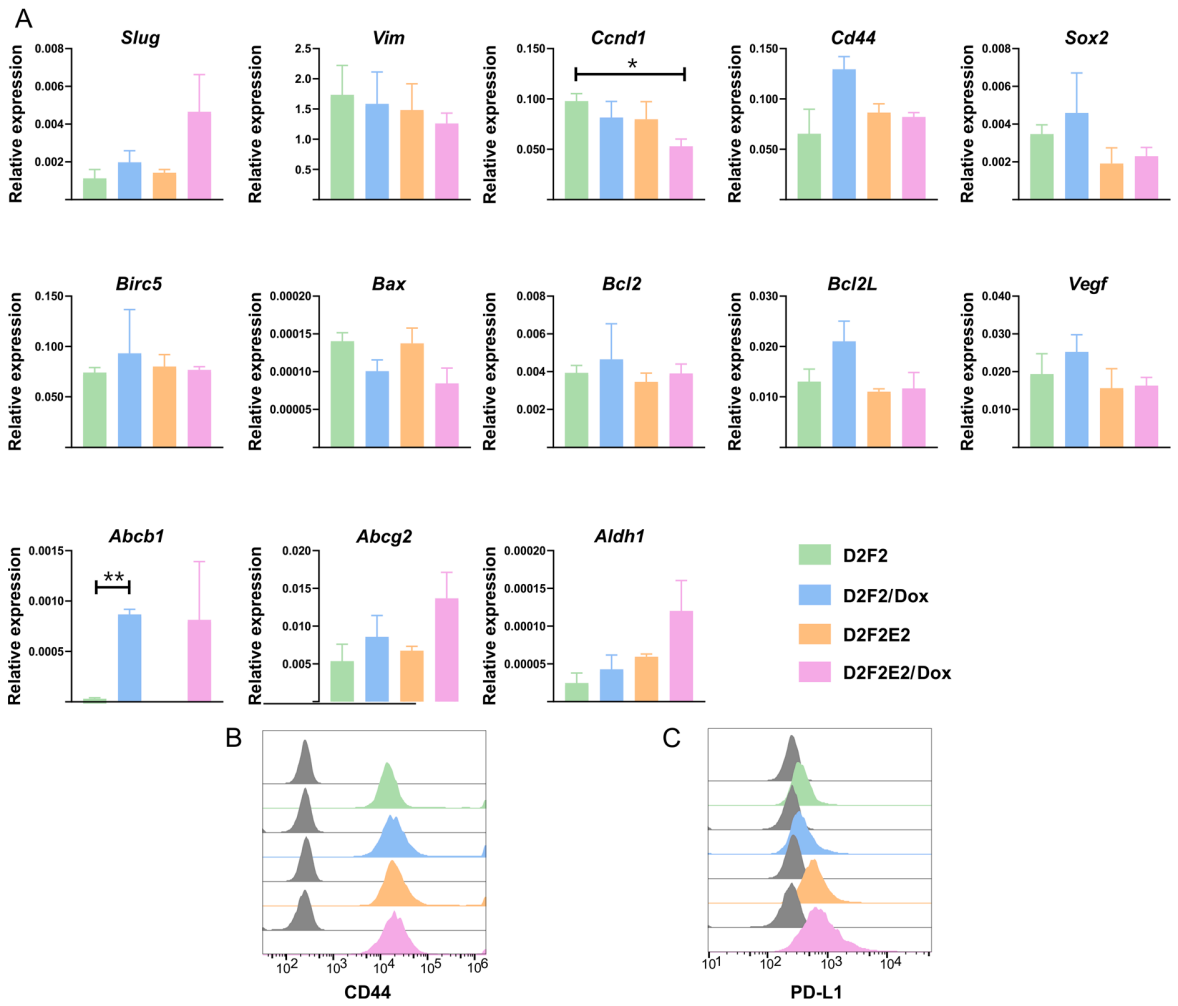
Expression of Stat3-depended genes and protein levels in chemotherapy-sensitive and -resistant breast cancer cells. (**A**) RNA was isolated from chemotherapy-sensitive and -resistant cancer cell lines, and gene expression was analyzed by qPCR. Gene expression level was normalized to GAPDH. The experiment was performed three times in duplicate. * Indicates *p* ≤ 0.05 and ** *p* ≤ 0.01. (**B**,**C**) Representative flow cytometry histograms showing the surface expression of CD44 (**B**) and PD-L1 (**C**) in D2F2, D2F2/Dox, D2F2E2, and D2F2E2/Dox cells. CD44 and PD-L1 were detected using APC-conjugated anti-CD44 and Alexa Fluor 647-conjugated anti-PD-L1 antibodies, respectively. Flow cytometry data were analyzed using FlowJo software (version 10.10.0; BD Biosciences).

The expression of genes related to Stat3 transcription factor, involved in the cancer-related processes, including oncogenesis, cell proliferation, metastasis, and apoptosis, was examined (Figure 4). The most distinguished difference in expression level was recognized in both types of Dox-resistant cells for *Abcb1* gene encoding a transporter protein that pumps drugs out of cells (Figure 4). For D2F2/Dox breast cancer cells the upregulation of *Abcb1* expression was significant comparing with drug-sensitive cell counterpart. Additionally, the Dox-resistant cells tended towards upregulation of the expression of *Abcg2*, encoding another transporter protein, and EMT related transcription factor *Slug.* Although the changes were not statistically significant, for both genes the upregulation was more pronounced in Dox-resistant D2F2E2 cells than in D2F2/Dox variant (Figure 4A). Moreover, the D2F2E2/Dox cells exhibited a significant decrease in the expression of cell proliferation-related gene *Ccnd1*. The *Aldh1* gene, which is frequently associated with chemoresistance and stemness in breast cancer, was also upregulated in chemotherapy-resistant variants, with the effect being more pronounced in cells exhibiting HER2 overexpression. The expression of the stemness-related *Cd44* gene was elevated in chemotherapy-resistant D2F2/Dox cells, whereas no significant change was observed in chemotherapy-resistant D2F2E2/Dox cells compared with their chemotherapy-sensitive counterparts. A very similar trend was observed for *Sox2* gene, the transcription factor related to the maintenance of stem cell pluripotency, and the *Vegf* gene, which is associated with angiogenesis. We also observed alterations in the expression of genes related to apoptosis. Although these changes did not reach statistical significance, they displayed a consistent trend. Specifically, the expression of the pro-apoptotic gene *Bax* was reduced in chemotherapy-resistant cells compared with their chemotherapy-sensitive counterparts. Additionally, the expression of anti-apoptotic *Birc5*, *Bcl2*, and *Bcl2L* genes were also slightly increased in D2F2/Dox cancer cells, with *Bcl2L* being the most upregulated. This reciprocal regulation pattern, despite lacking statistical confirmation, suggests a potential shift in the apoptotic balance of chemotherapy-resistant cells, especially in D2F2/Dox variant, which may reflect adaptive changes in cell survival mechanisms under the selective pressure of Dox (Figure 4A).

In addition, the levels of cell-surface markers associated with stemness were assessed by flow cytometry. CD44 was detected at high levels on the surface of all analyzed cell lines; however, differences were observed in the mean fluorescence intensity (MFI) between cell variants. Consistent with the mRNA expression data, D2F2/Dox cells exhibited higher CD44 surface presence than their chemosensitive D2F2 counterparts (MFI 18,900 vs. 14,530, respectively) (Figure 4B). In contrast, acquisition of doxorubicin resistance did not alter CD44 expression levels in D2F2E2 cells, as reflected by comparable MFI values between D2F2E2 and D2F2E2/Dox cells (19,721 vs. 19,460, respectively) (Figure 4B). The analysis also confirmed slightly higher CD44 presence on D2F2E2 cells than on D2F2 counterpart. Furthermore, differences in PD-L1 level were observed between chemosensitive and chemoresistant cell populations. The percentage of PD-L1-positive cells increased following the acquisition of doxorubicin resistance (17.6% vs. 23.6% and 60.7% vs. 71.9%, for D2F2 vs. D2F2/Dox and D2F2E2 vs. D2F2E2/Dox, respectively) (Figure 4C). Additionally, HER2-overexpressing cells exhibited a higher percentage of PD-L1-positive cells compared with cells lacking HER2 overexpression (Figure 4C). It should also be noted that the observed changes in CD44 and PD-L1 expression are consistent with stem-like or cancer stem cell-related characteristics; however, these markers alone do not establish a functional cancer stem cell phenotype.

Similar alterations in gene expression driven by STAT3 activity or associated with chemoresistance have been reported in a wide range of cancer research. A study by Zhang et al. suggests that activated STAT3 directly interacts with the *ABCB1* promoter, thereby stimulating its expression in leukemia [71]. Moreover, a similar STAT3–ABCB1 regulatory relationship has been observed in chemotherapy-resistant hepatocellular carcinoma [72]. A key role of ABCB1 is in chemotherapy resistance, where its overexpression in cancer cells can pump out anti-cancer drugs. However, ABCB1, beyond its canonical role in mediating chemoresistance via ATP-dependent drug efflux, also plays a critical role in regulating and maintaining cancer stemness [73]. Consistent with evidence linking ABC transporters to cancer stem cells, *ABCB1* is preferentially expressed in stem-like tumor subpopulations, where it supports an undifferentiated state, self-renewal capacity, and long-term tumor-initiating potential. Through its ability to protect cells from cytotoxic stress and to interface with stemness-associated signaling programs, ABCB1 enables CSCs to survive chemotherapy and adapt to hostile microenvironmental conditions. This functional coupling of multidrug resistance and stemness confers a selective survival advantage, promoting tumor progression, therapeutic failure, and disease relapse [73]. ABCG2, a member of the ATP-binding cassette transporter family, also functions as a drug-efflux pump and plays a key role in multidrug resistance by reducing intracellular accumulation of chemotherapeutic agents [74]. Literature evidence indicates a link between STAT3 activation and ABCG2 upregulation in the context of cancer stem cell–associated chemoresistance. Tumor-associated macrophages induce a CSC-like phenotype in breast cancer cells by upregulating the EGFR/STAT3/SOX2 signaling axis, which promotes ABCG2 expression and enhances drug efflux capacity [75]. Elevated ABCG2 levels have also been observed in chemo-treated breast CSCs as part of a SOX2/ABCG2/TWIST1 pluripotency–chemoresistance–EMT network [76]. Furthermore, breast cancer side population cells, defined by high *ABCG2* expression, display increased resistance to mitoxantrone and are enriched in triple-negative tumors [77]. Together, these findings support a functional correlation between STAT3 signaling and ABCG2-mediated drug efflux, contributing to therapy resistance in breast cancer.

As mentioned above, one possible molecular mechanism of cell chemoresistance is through the acquisition of the stemness phenotype. In breast cancer, ALDH1 is predominantly associated with the induction of cancer stem cell (CSC) properties and chemoresistance in tumor cells [78]. Its expression and functional activity have been shown to be dependent on STAT3 signaling, highlighting a key regulatory axis in the maintenance of stem-like traits [79]. Furthermore, ALDH1 has been linked to CD44, a well-established marker of CSCs, suggesting a coordinated role in promoting stemness and therapeutic resistance within the tumor cells [78,79]. In breast cancer, a strong association between CD44 expression and STAT3 activation has been consistently observed in cancer stem cell population [32,80]. The CD44+/high/CD24−/low breast cancer cell subpopulation, which exhibits enhanced stem-like properties such as mammosphere formation, also displays elevated STAT3 expression and phosphorylation, contributing to chemoresistance, including resistance to tamoxifen and doxorubicin [31,80]. Notably, inhibition or silencing of STAT3 resulted in a significant reduction in CD44 expression, suggesting that STAT3 is a key mediator of CD44 expression [79]. SOX2 is identified as a key driver of both stemness and chemoresistance in cancer. SOX2, a transcription factor central to pluripotency and self-renewal, is highly expressed in breast cancer stem cell (CSC) populations that survive paclitaxel treatment [76]. Elevated SOX2 correlates with enhanced sphere-forming capacity, increased expression of stem cell markers, and resistance to chemotherapy, while silencing SOX2 reduces CSC self-renewal and sensitizes cells to chemotherapeutic drugs [50]. Vascular Endothelial Growth Factor (VEGF) is a key mediator of angiogenesis, promoting endothelial cell proliferation, migration, and vascular formation, which are critical processes in tumor growth and progression [81]. STAT3 can stimulate VEGF expression, linking this transcription factor to angiogenic regulation in cancer [82]. In breast cancer, VEGF activity has also been associated with the maintenance of stem-like properties in cancer cells, supporting their self-renewal and tumorigenic potential [83]. Consequently, VEGF may contribute to chemoresistance associated with cancer stem cell phenotypes, underscoring its potential as a therapeutic target to overcome treatment-resistant tumors.

The interplay between Snail family transcription factors, STAT3, and therapy resistance emerges as a recurrent theme across multiple cancer types. In endocrine-resistant ER+ breast cancer, SNAI2 (Slug) is markedly upregulated, driving epithelial–mesenchymal transition, increasing cell motility, and promoting an aggressive phenotype. Moreover, its high expression correlates with shorter progression-free survival under endocrine therapy [84]. Similarly, in non-small cell lung cancer, the CXCR4/STAT3/Slug axis enhances resistance to ionizing radiation by promoting EMT and reducing DNA damage accumulation [85]. In ovarian cancer, phosphorylated STAT3 (Y705) promotes metastasis and cisplatin resistance by regulating Slug-mediated E-cadherin and Vimentin, in conjunction with MAPK and PI3K/AKT pathway activation, further linking STAT3–Slug signaling to both EMT and therapy resistance [86]. Interestingly, in our model, higher Slug expression correlated with enhanced motility in Dox-resistant cells. In breast cancer, elevated expression of *Slug* has been directly linked to increased cellular motility. In triple-negative breast cancer models, Slug represses the cell–cell adhesion protein plakoglobin, and overexpression of Slug in Slug-deficient cells significantly increases migration, whereas knockdown of *Slug* reduces motility, indicating that Slug-mediated transcriptional repression enhances the migratory capacity of aggressive breast cancer cells [87]. In ovarian carcinoma models, chemoresistant cells exhibit increased *Slug* expression along with other EMT markers [63]. By reducing *Slug* (and *Snail*) expression, the cells were re-sensitized to cisplatin, indicating a direct role of *Slug* in drug resistance [88]. Similarly, in head and neck squamous cell carcinoma (HNSCC), *Slug* overexpression enhances stem-like traits, increases resistance to cisplatin, and promotes invasion, whereas *Slug* knockdown reduces stemness and chemoresistance, suggesting that *Slug* is a potential therapeutic target to overcome treatment resistance in CSC-like HNSCC cells [89].

Constitutive activation of STAT3 plays a pivotal role in regulating the balance between pro- and anti-apoptotic BCL-2 family members, thereby contributing to therapy resistance in cancer. In metastatic estrogen receptor-negative breast cancer, STAT3 activation is associated with elevated BCL-2 levels, which promote cell survival and reduce chemotherapy-induced apoptosis [90]. This anti-apoptotic influence is reinforced by STAT3-mediated suppression of BAX, a pro-apoptotic protein, shifting the BAX:BCL-2 ratio toward a survival-favoring state [91]. Since BCL-2 can neutralize BAX activity through heterodimer formation, this imbalance effectively inhibits mitochondrial apoptotic signaling, supporting the persistence of resistant cancer cell populations. Collectively, these findings highlight the STAT3–BCL-2–BAX axis as a key determinant of apoptosis evasion and therapeutic resistance in breast cancer [91].

Cyclin D1 (CCND1) is a key regulator of the G_1_–S phase transition in the cell cycle, acting by phosphorylating the retinoblastoma protein to stimulate proliferation [92]. Beyond its canonical cell cycle function, CCND1 also participates in CDK-independent processes, including transcriptional regulation, chromatin remodeling, metabolic control, adipogenesis, and cell migration [92]. Elevated expression of *CCND1* has been reported in tamoxifen-resistant breast cancer, supporting sustained proliferation despite estrogen receptor antagonism [93]. Mechanistically, STAT3 frequently acts as an upstream activator of *CCND1* transcription in cancer, and its constitutive activation is linked to increased CCND1 levels and oncogenic transformation [94]. Interestingly, in our doxorubicin-resistant breast cancer model, *CCND1* expression was downregulated, especially in the D2F2E2/Dox cells, which may correlate with their reduced proliferative capacity. As mentioned above, one possible molecular mechanism of cell chemoresistance is through the acquisition of the stemness phenotype. Cancer stem cells often proliferate much more slowly or are quiescent, which can explain their resistance to chemotherapeutic drugs that target rapidly proliferating cancer cells [95]. Recent reports indicate that CCND1 plays a dual role, as its action may promote or inhibit cell differentiation, senescence, apoptosis, or cell cycle progression, depending on the cellular and molecular context [96].

It is worth noting that D2F2 and D2F2E2 breast cancer cells also differ in their gene expression profiles. The overexpression of *the ERBB2/neu* gene encoding HER2 resulted in a slight increase in *Stat3*, and especially in *Cd44* and *Aldh1* gene expression (Figure 3A and Figure 4), indicating a shift into stemness characteristics. It was shown that HER2 overexpression promotes cancer stem cell (CSC) properties, enhancing self-renewal and stemness signaling, which contributes directly to chemoresistance [97]. HER2 drives expansion of the mammary stem/progenitor population, increasing mammosphere formation and CSC marker expression such as ALDH, thereby enriching cells capable of surviving cytotoxic therapies [98]. It also activates stemness-associated pathways, including Notch and Wnt/β-catenin, which reinforce CSC traits and protect HER2-positive cells from chemotherapy and targeted therapies such as trastuzumab [39]. As a result, HER2-enriched CSC-like populations exhibit enhanced tumor initiation, invasiveness, and drug resistance, linking HER2 signaling mechanistically to both tumor aggressiveness and chemoresistance [97].

Increased PD-L1 expression has been reported in cancer cells that acquire chemoresistance, suggesting that PD-L1 upregulation may be associated with cellular adaptation to prolonged therapeutic stress [99]. Several studies have demonstrated that chemotherapy-resistant cell populations exhibit higher PD-L1 levels compared with parental, drug-sensitive cells, indicating that PD-L1 expression may be linked to the acquisition of a more aggressive and therapy-resistant phenotype [100,101]. For example, Zhang et al. observed increased PD-L1 expression in non-small cell lung cancer following chemotherapy exposure, suggesting that treatment-induced cellular changes may contribute to PD-L1 regulation [100]. Similarly, elevated PD-L1 expression has been described in HER2-overexpressing cancer cells. In HER2-positive breast cancer, PD-L1 levels have been associated with HER2 status, suggesting a potential connection between HER2-driven signaling and regulation of PD-L1 expression [102,103].

Based on the results of this study, supported by evidence from the literature, including gene expression profiles and analysis of the Stat3 state, cellular proliferation, migration, and morphology, we established the chemotherapy-resistant D2F2/Dox and D2F2E2/Dox breast cancer cell lines. The proposed mechanism of chemoresistance can be associated with the acquisition of stem-like properties, while modulation of apoptotic pathways also plays a significant contributing role. Overexpression of HER2 in breast cancer cells slightly induced stem-like features, and continuous drug treatment further enhanced these features, as evidenced by a trend towards higher expression of *Aldh* and *Slug* genes. Additionally, drug pressure increased the level of phosphorylated Stat3 at Y705 and S727, indicating high Stat3 activation in D2F2E2/Dox cells. In the D2F2 breast cancer cell line, in addition to a slight upregulation of stem-related genes, doxorubicin treatment appears to have a more profound effect on genes controlling apoptosis. Although the phosphorylation status of Stat3 also increased in D2F2/Dox cells, it affected only Y705 and was not as strong as in D2F2E2/Dox cells. These data indicate that the acquired doxorubicin resistance was associated with Stat3 status; however, HER2- and HER2+ breast cancer cell lines did not indicate the same mechanism of chemoresistance acquisition.

### 3.5. Analysis of the Expression of Genes Related to the Stat3 Transcription Factor in Dox-Resistant Breast Cancer Cells After Stat3 Silencing

**Figure 5:**
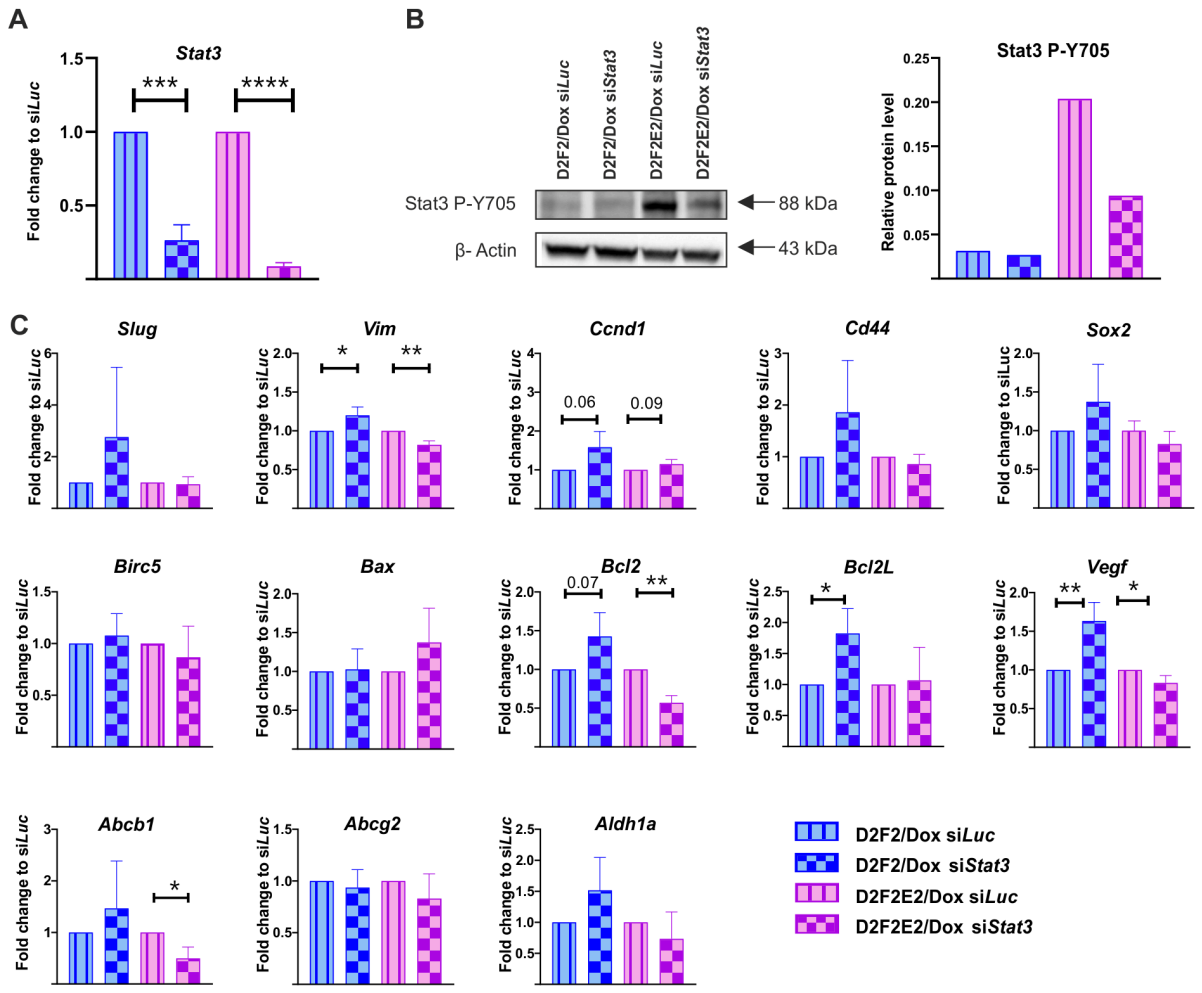
Effect of Stat3 silencing on the Stat3 mRNA and protein level and the expression of Stat3-related genes in chemotherapy-resistant breast cancer cells. D2F2/Dox and D2F2E2/Dox cells were treated with siStat3-siRNA (or control siLuc-siRNA) for 48 h, then the following analyses were performed. (**A**) RNA was isolated and the expression of (**A**) Stat3 gene and (**C**) Stat3-related genes were analyzed by qPCR. Expression level was normalized to GAPDH and presented as fold change relative to the siLuc control for each cell line. Experiments were performed three times in duplicate. Data were analyzed using GraphPad Prism. * Indicates *p* ≤ 0.05, ** *p* ≤ 0.01, *** *p* < 0.001, and **** *p* < 0.0001. (**B**) Protein was extracted from treated cells in the presence of protease and phosphatase inhibitors, and Western blot analysis was performed to compare Stat3 P-Y705 protein level. Changes in protein expression levels were determined by densitometric analysis and normalized to β-actin as a loading control. Densitometric quantification of representative Western blot bands is shown as relative intensity normalized to β-actin. The data from one experiment is shown.

To examine the hypothesis regarding the role of Stat3 in the development of drug resistance in breast cancer cells, we additionally silenced its expression using siRNA in Dox-resistant cell variants. First, the *Stat3* knockdown was confirmed in both Dox-resistant cell types at the mRNA level (Figure 5A). Additionally, silencing *Stat3* resulted in decreasing the level of Stat3 phosphorylated at Y705 in D2F2/Dox and D2F2E2/Dox cells, with a much higher impact in D2F2E2/Dox breast cancer cells (Figure 5B).

The silencing of *Stat3* in D2F2/Dox and D2F2E2/Dox led to the regulation of many gene expressions (Figure 5C). Specifically, *Slug* expression was upregulated in D2F2/Dox cells following *Stat3* silencing, whereas no changes were observed in D2F2E2/Dox cells. *Ccnd1* expression was elevated in both D2F2/Dox and D2F2E2/Dox cells, a pattern opposite that observed in chemotherapy-resistant cells compared with their sensitive counterparts. *Vegf* expression was upregulated in D2F2/Dox cells, whereas it was downregulated in D2F2E2/Dox cells. *Vim* expression was markedly reduced in D2F2E2/Dox cells, whereas it was elevated in D2F2/Dox cells. A similar pattern was also observed for other stemness-associated genes, such as *Cd44*, *Aldh1*, and *Sox2*; however, these changes did not reach statistical significance. In the expression profile of genes involved in apoptosis regulation, the following changes were observed upon *Stat3* silencing: the anti-apoptotic genes *Bcl2* and *Bcl2L* were upregulated in D2F2/Dox cells, whereas in D2F2E2/Dox cells, only *Bcl2* showed significant downregulation. A similar trend was also noted for the *Birc5* gene, with an upregulation in D2F2/Dox cells and downregulation in D2F2E2/Dox cells. In contrast, the pro-apoptotic gene *Bax* was upregulated in both cell lines, especially in the *Stat3*-silenced D2F2E2/Dox breast cancer cells. Finally, genes encoding transporter proteins also exhibited alterations following *Stat3* silencing. *Abcg2* expression showed a slight decrease in both cell lines, whereas *Abcb1* expression was markedly reduced in D2F2E2/Dox cells but increased in D2F2/Dox cells (Figure 5C).

We carefully analyzed the genes in which silencing of Stat3 resulted in an expression pattern opposite to that associated with the acquisition of chemoresistance. The chemotherapy-resistant breast cancer cells showed increased *Stat3* expression, which was associated with altered expression patterns of other genes. After *Stat3* silencing in Dox-resistant cells, the expression pattern of these genes should indicate a tendency to revert to their previous levels if their expression were Stat3-dependent. Indeed, we observed that *Stat3* silencing exerted this effect; however, the effect varied depending on the cells’ HER2 status. Such a pattern was more frequently detected in cells with HER2 overexpression and was evident for genes such as *Abcb1*, *Aldh1*, *Abcg2*, *Bax*, *Bcl2*, *Ccnd1*, and *Sox2* (Figure 5C). This finding may suggest a significant role for HER2 and Stat3, as well as their potential interdependence, in controlling the expression of the analyzed genes and possibly in the development of chemoresistance. Interestingly, many of the reverse-regulated genes are involved in the acquisition of the stemness phenotype, suggesting that *Stat3* silencing could reduce the stem-like properties of HER2-overexpressing Dox-resistant breast cancer cells. Additionally, the *Bax:Bcl2* ratio shifted toward a more apoptotic-favoring state in *Stat3*-silenced D2F2E2/Dox cells, accompanied by a substantial reduction in the levels of genes encoding transporter proteins. All these evidences suggest the potential reduction in chemoresistant state in *Stat3*-silenced D2F2E2/Dox cells.

Interestingly, the *Stat3*-silenced D2F2/Dox cells exhibited a distinct pattern of gene expression compared to the HER2-overexpressing cell variant. For D2F2/Dox cells, only *Vim*, *Ccnd1*, and slightly *Abcg2* indicated a reverse expression pattern after *Stat3* silencing. Surprisingly, based on gene expression analysis in *Stat3*-silenced D2F2/Dox cells, there appears to be a trend toward increased stemness potential and further enhancement of apoptotic evasion (Figure 5C). Previously, it was indicated that silencing of STAT3 in cancer cells significantly increased the NFκB transcriptional activity under basal conditions [104] and NFκB induces the expression of genes involved in inflammation and apoptosis, including *cIAP2*, *Bcl2*, and *BclxL* [105]. Thus, in our model, silencing *Stat3* in D2F2/Dox cells could increase expression of *Bcl2* and Bcl2L (*BclxL*) through more activated NFκB. However, it needs further study.

### 3.6. Functional Assays Following Stat3 Silencing

**Figure 6:**
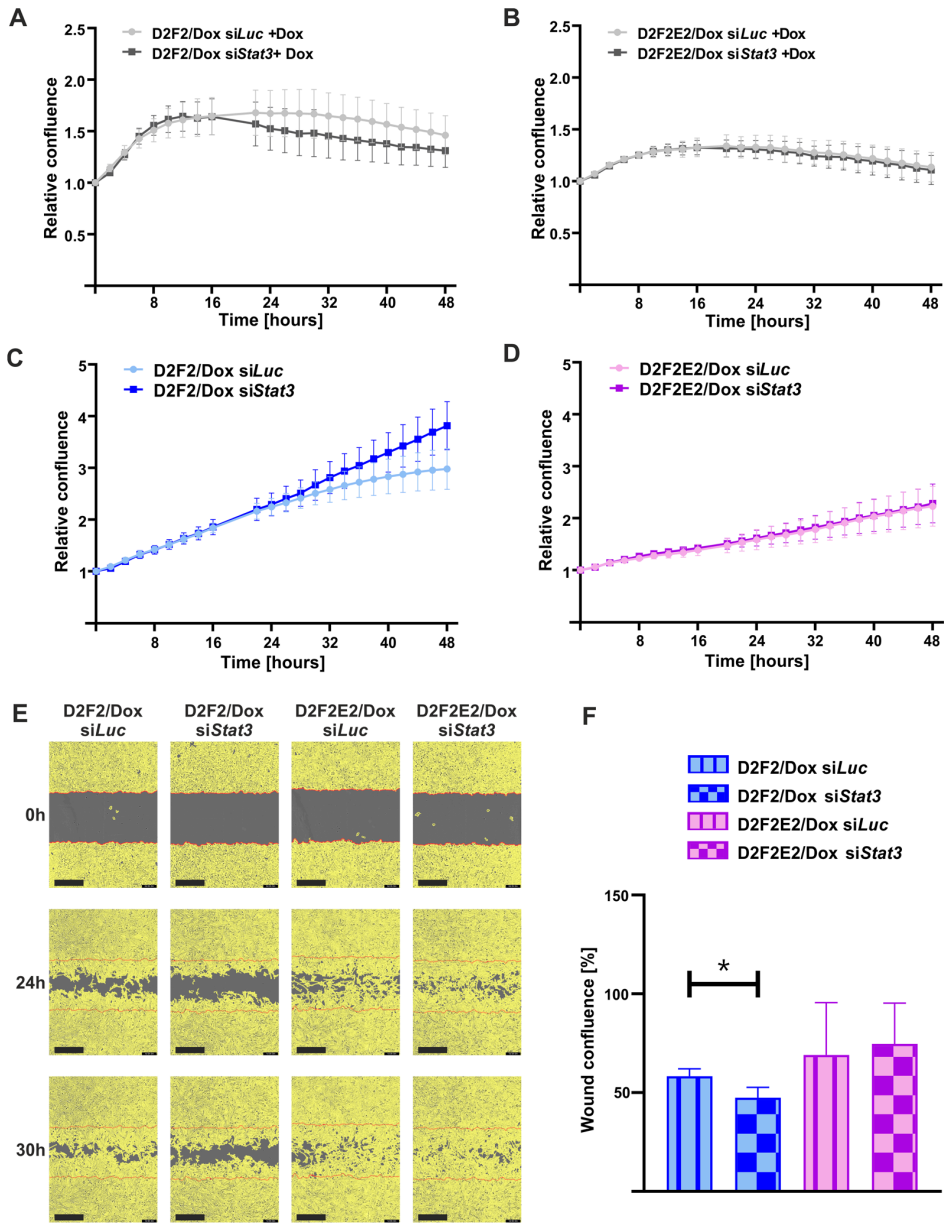
Functional effects of *Stat3* silencing on chemotherapy-resistant cells. D2F2/Dox and D2F2E2/Dox cells were treated with *Stat3*-targeting siRNA (or control *Luc*-targeting siRNA) for 48 h and subsequently seeded in 96-well plates for further assays. (**A**,**B**) Cell confluence in the presence of 64 µg/mL doxorubicin was assessed in real-time using IncuCyte. (**C**,**D**) Cell proliferation was monitored in real-time by measuring confluence using the IncuCyte system. (**E**,**F**) Cell migration was evaluated by measuring the wound healing rate in real-time using the IncuCyte system. (**E**) Representative images of the wound healing assay at 0, 24, and 30 h. Scale bar 400 μm (bottom left). (**F**) Wound Confluence after 24 h. Each experiment was performed three times in duplicate (mean ± SEM). Data were analyzed using IncuCyte software and GraphPad Prism. * Indicates *p* ≤ 0.05.

Based on alterations in the gene expression profile of chemotherapy-resistant cells following *Stat3* silencing, we performed functional assays targeting various cellular processes. First, the cells’ sensitivity to doxorubicin was analyzed. Upon exposure to doxorubicin at a concentration of 64 µg/mL, the number of cells was lower in the D2F2/Dox si*Stat3* group than in the control group. However, this difference was not statistically significant (Figure 6A). These findings may suggest that D2F2/Dox si*Stat3* cells exhibit a trend in reduced tolerance to doxorubicin and, consequently, reduced chemoresistance. The experiment was repeated in three biological repetitions indicated in Supplementary Figure S1. Due to differences in confluence level between biological repetitions, the mean value indicates high scatter of results; however, in the each repetition, the trend was the same, indicating slightly higher drug sensitivity of D2F2/Dox si*Stat3* comparing with control D2F2/Dox si*Luc* cells (Supplementary Figure S1). In the D2F2E2/Dox groups, the number of cells did not differ between the *Stat3*-silenced and control groups (Figure 6B).

Moreover, the proliferation of D2F2/Dox cells increased following *Stat3* silencing; however, this change did not reach statistical significance (Figure 6C, Supplementary Figure S1). The trend of increased proliferation in D2F2/Dox si*Stat3* cells correlated with higher *Ccnd1* expression. Moreover, it indicates that the lower number of D2F2/Dox si*Stat3* cells after Dox treatment is due to the drug’s higher cytotoxicity rather than a reduced ability of the cells to grow. In D2F2E2/Dox cells, *Stat3* silencing had no detectable effect on proliferation (Figure 6D, Supplementary Figure S1). Additionally, the migration of D2F2E2/Dox si*Stat3* cells remained unchanged compared to the controls (Figure 6E,F) By comparison, the migration of D2F2/Dox cells after *Stat3* silencing was significantly reduced (Figure 6E,F), which was rather surprising, since the gene expression analysis indicated a trend of higher expression level of *Slug* or *Vim* (Figure 5C).

In our model, we did not observe a significant reduction in chemoresistance upon *Stat3* silencing, although decreases in Stat3 expression and phosphorylation were observed, especially in D2F2E2/Dox cells. The trend of increased drug sensitivity was marginally indicated in D2F2/Dox si*Stat3* cells; however, it was inconsistent with the results showing that anti-apoptotic genes were upregulated in these cells after silencing Stat3 (Figure 5). Interestingly, Kulesza et al. indicated that *STAT3* depletion increased the NFκB activity in cancer cells providing the compensatory, pro-survival signal; however, the doxorubicin treatment resulted in the decrease in the NFκB activity in these cells compared to the control cells [104]. The similar relation could occur in our model, but we did not analyze the expression and activity of various factors after *Stat3* silencing and Dox treatment. The lack of significant changes following *Stat3* silencing may be attributed to the presence of alternative mechanisms sustaining chemoresistance in these cells that were not investigated in the present study. It is also possible that the alterations detected at the gene expression level were insufficient in magnitude to exert a measurable impact on the cellular processes. Moreover, suppression of STAT3 rarely resulted in a complete restoration of drug sensitivity in breast cancer cells. Instead, the most commonly observed effect is a reduction in the IC_50_ value for the drug to which the cells have acquired resistance; nevertheless, drug sensitivity typically does not return to the level observed in the parental, treatment-naive cell population [32,106,107]. STAT3 has also been proposed as a component of combination therapeutic strategies, where its modulation may potentiate the reversal of chemoresistance achieved by other interventions [107]. For instance, in MCF-7/Dox resistant cells, the *STAT3* silencing exerted minimal effect in resensitizing cells to doxorubicin; however, simultaneously silencing of *STAT3* and *P65* (NF-κB) had the strong ability to reverse resistant cells (higher that silencing only *P65*) [107]. It suggests that targeting STAT3 may need synergistic others molecular approaches aimed at disrupting resistance-associated signaling pathways.

### 3.7. Targeted Delivery of siStat3

Moreover, we investigated whether spider silk-based nanoparticles (NP) could serve as carriers for si*Stat3* delivery. For this purpose, we employed H2.1MS1:MS2KN silk spheres, which had previously been developed for the delivery of siRNA into cell lines overexpressing HER2 [51,54]. These nanospheres were functionalized with the H2.1 peptide, for HER2 targeting, and the KN peptide, which enhances nucleic acid binding. siRNA was loaded into the nanospheres and administered to chemotherapy-resistant cells with or without HER2 overexpression [54].

**Figure 7:**
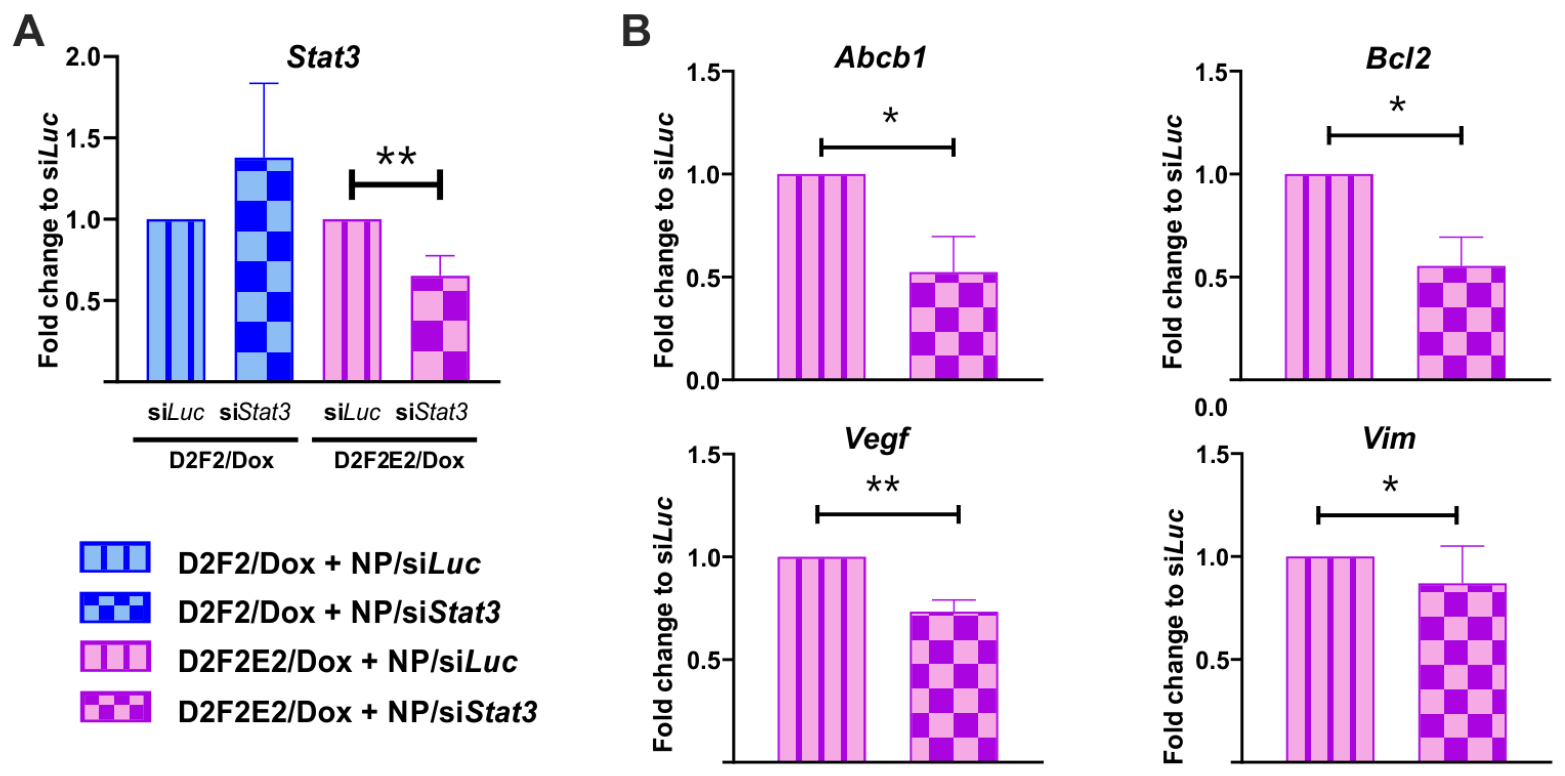
Effect of *Stat3* silencing after siRNA *Stat3* delivery via H2.1MS1:MS2KN silk spheres in chemotherapy-resistant breast cancer cells. (**A**) D2F2/Dox and D2F2E2/Dox cells were treated with H2.1MS1:MS2KN spheres (NP) loaded with si*Stat3* (or control si*Luc*) for 48 h. RNA was then isolated, and *Stat3* expression was analyzed by qPCR. (**B**) D2F2E2/dox cells were treated with H2.1MS1:MS2KN spheres loaded with si*Stat3* (or control si*Luc*) for 48 h. RNA was then isolated, and the expression levels of *Abcb1*, *Bcl2*, *Vegf*, and *Vim* were analyzed by qPCR. Expression levels were normalized to GAPDH and are presented as fold change relative to the si*Luc* control for each cell line. The experiment was performed three times in duplicate. Data were analyzed using GraphPad Prism. * Indicates *p* < 0.05 and ** *p* ≤ 0.01.

Using this approach, we achieved *Stat3* silencing in HER2-overexpressing cells; however, no silencing was observed in cells lacking HER2 overexpression (Figure 7A). The extent of silencing was approximately 45%, which is significantly lower than that achieved for si*Stat3* delivery using Lipofectamine (~90%). In our previous study, *Stat3* silencing using H2.1MS1:MS2KN spheres in ovarian cancer cells SKOV3 was markedly more effective (70%) [54]. This discrepancy may be due to differences in HER2 levels between cell lines. Despite the lower overall efficiency of *Stat3* silencing when delivered via spheres, the changes in expression of Stat3-dependent genes were comparable to (*Abcb1*, *Bcl2*, *Vim*) or even more pronounced (*Vegf*) than those observed with Lipofectamine-mediated siRNA delivery (Figure 7B vs. Figure 5C).

The HER2-targeted delivery of siRNA via nanospheres appears to be a more favorable approach, particularly in in vivo studies. First, this approach enables the selective delivery of si*RNA* to HER2-overexpressing cells, thereby minimizing potential adverse effects on both HER2-negative cells, as indicated in this study, and healthy cells. Due to its functional role, STAT3 presents a dual challenge: on one hand, it acts as an oncogene and thus constitutes a crucial therapeutic target in cancer treatment; on the other hand, it is also essential for the proper functioning of healthy tissues. For example, *Stat3* silencing in cardiomyocytes has been shown in mice to increase susceptibility to inflammation, promote cardiac fibrosis, and lead to age-associated heart failure [108]. Similarly, keratinocyte-specific Stat3 ablation results in impaired keratinocyte migration, delayed wound healing, and defects in hair and ulcer repair, highlighting its critical role in normal skin remodeling [109]. Second, the application of H2.1MS1:MS2KN spheres eliminates the need for the transfection reagent Lipofectamine, which can be toxic and is unsuitable for in vivo applications [110,111]. Moreover, the nanospheres protect siRNA from endonuclease degradation, which may prolong its availability and functional activity in the cells [112]. In summary, the H2.1MS1:MS2KN spheres, as a drug-delivery tool, can provide targeted delivery, reduce off-target side effects, enhance drug bioavailability, and decrease dosing frequency, thereby improving the overall efficacy of siRNA-based drugs. However, it requires further advanced study in an in vivo setting.

## 4. Conclusions

In this study, we established a doxorubicin-resistant model in HER2- and HER2+ breast cancer cells. The chemotherapy-resistant D2F2/Dox and D2F2E2/Dox cells differed from the parental cells and from each other in terms of cellular morphology, proliferation, and migration. In both cell lines, chemoresistance was correlated with elevated levels of Stat3 at both the mRNA and activated protein levels, suggesting that this may be a key determinant. We analyzed the expression of Stat3-dependent genes and found that, in both cell lines, chemoresistance arose predominantly through mechanisms associated with enhanced cellular stemness and increased drug efflux capacity, although modulation of apoptotic pathways also contributed significantly, especially in D2F2/Dox cells.

To confirm the Stat3 involvement, we silenced *Stat3* in doxorubicin-resistant cells. Although we observed that *Stat3* silencing exerted a more pronounced effect in HER2-overexpressed cells, reversing the expression of genes involved in drug efflux, stemness, and apoptosis, the functional assays did not indicate any change in cellular behavior. It may be related to HER2 status, since HER2 signaling contributes to the regulation of *STAT3* expression [40,113] and to reduced drug sensitivity [12,13,14,15,16,17,18]. In our model, the higher level of phosphorylated *Stat3* was also detected in parental, chemotherapy-sensitive, HER2-overexpressing cells relative to HER2-negative controls, indicating a baseline activation of this pathway associated with HER2 status. The parental D2F2E2 cells also showed an increased level of *Cd44* and *Aldh1*, suggesting a more stem-like phenotype than in the D2F2 group. Thus, silencing *Stat3* may be insufficient, and a combinatory therapy targeting other pathways may be the next step. Simultaneous inhibition of HER2 signaling, suppression of Stat3 and other factors that regulate the stemness program and drug efflux mechanism may collectively overcome compensatory resistance networks that limit single- or dual-modality treatments. However, we acknowledge that, in our study, we did not fully investigate the functional cancer stem cell phenotype of the cells examined.

In contrast, resistance development in D2F2/Dox cells appeared to involve another mechanism. Although *Stat3* expression and phosphorylation were increased in chemoresistant D2F2 cells, after efficient silencing of *Stat3* at the mRNA level, the phosphorylated Stat3 levels in D2F2/Dox cells remained unchanged. It suggests that Stat3 activation, rather than total expression, is critical for maintaining the resistant phenotype. Additionally, after *Stat3* silencing, a few genes showed a reversal in expression compared to before chemoresistance acquisition. In contrast, gene expression analysis indicates that these cells are prone to shift toward a more mesenchymal, stem-like, and reduced apoptosis phenotype. These changes did not reach the statistical significance; however the trend was repetitive. At that point, this phenomenon is unclear. On the other hand, functional studies showed that silencing of *Stat3* significantly reduced the migratory potential of these cells. The significant shift in migratory potential was observed twice: once after acquisition of chemoresistance (a marked increase in migration in D2F2/Dox compared with D2F2 cells) and second time after *Stat3* silencing (a significant decrease in migration in D2F2/Dox si*Stat3* compared with D2F2/Dox si*Luc* cells). These data indicate that Stat3 was more closely associated with the migratory potential of chemoresistant cells than with the direct acquisition of chemoresistance. The mechanism of chemoresistance in D2F2/Dox cells may involve alternative mechanisms that were not investigated in the present study.

Importantly, our study also demonstrates the applicability of a proprietary targeted nucleic acid-based drug delivery system designed for selective molecular intervention in HER2-overexpressing cells. This system, based on functionalized silk spheres, enabled efficient intracellular delivery of nucleic acids and effective knockdown of *Stat3* and its downstream target genes. The DDS system based on H2.1MS1 and other silk variants with various functionalization can enable targeted delivery of diverse therapeutic molecules to specific cell types [52,114,115,116]. Simultaneous action of a variety of therapeutics targeting distinct processes related to chemoresistance, delivered precisely via silk-based DDS, may collectively restore drug sensitivity in cells.

Although the D2F2E2 model is a valuable tool for investigating HER2-driven mechanisms in the context of doxorubicin adaptation, it has some limitations. D2F2E2 cells are of murine origin and express human HER2 through genetic modification, resulting in constitutive HER2 overexpression that may not fully reflect the dynamic regulation and heterogeneity of HER2 expression observed in human tumors; the D2F2E2 cells constitute a mouse model modified to overexpress human HER2 and are not necessarily directly equivalent to a human HER2+ clinical subtype. In addition, an empty-vector control line was not included in the present study. Therefore, potential effects associated with plasmid integration and antibiotic selection cannot be completely excluded, and direct comparisons between parental D2F2 and D2F2E2 cells should be interpreted with caution. Importantly, the primary aim of the present study was not to directly compare the parental D2F2 and D2F2E2 cell lines, but rather to investigate doxorubicin resistance-associated changes within each cellular background, comparing D2F2 with D2F2/Dox and D2F2E2 with D2F2E2/Dox.

Nevertheless, the controlled nature of this model allows the contribution of HER2 overexpression to therapy adaptation to be studied under defined experimental conditions. The developed doxorubicin-resistant HER2- and HER2+ breast cancer model may be used to analyze potential therapy targets and drug delivery methods. Further investigation in future studies, including additional independent validation of the observed protein-level changes and analysis of a broader panel of signaling and cell death-related proteins, may provide a more comprehensive understanding of the molecular mechanisms underlying doxorubicin adaptation in these models.

## Figures and Tables

**Table 1:** List of primer sequences used for qPCR.

Gene	Forward Primer	Reverse Primer
*Gapdh*	5′-TCACTGCCACCCAGAAGACT-3′	5′-ATGCCAGTGAGCTTCCCGTT-3′
*Stat3*	5′-GGAATAACGGTGAAGGTGCT-3′	5′-CATGTCAAACGTGAGCGACT-3′
*Birc5*	5′-TACCGAGAACGAGCCTGATT-3′	5′-CAGGGGAGTGCTTTCTATGC-3′
*Bcl2*	5′-TGGGGATGACTTCTCTCGTC-3′	5′-TCAAAGAAGGCCACAATCCT-3′
*Bcl2L1*	5′-TGGACAATGGACTGGTTGAG-3′	5′-GGGGCCTCAGTCCTATTCTC-3′
*Bax*	5′-CACGTCCACGATCAGTCAC-3′	5′-CCTGGATGAAACCCTGTAGC-3′
*Ccnd1*	5′-TTGACTGCCGAGAAGTTGTG-3′	5′-CCACTTGAGCTTGTTCACCA-3′
*Vim*	5′-GATCAGCTCACCAACGACAA-3′	5′-TCCACTTTCCGTTCAAGGTC-3′
*Sox2*	5′-GCGGAGTGGAAACTTTTGTCC-3′	5′-GGGAAGCGTGTACTTATCCTTCT-3′
*Slug*	5′-GGCTGCTTCAAGGACACATT-3′	5′-GATGTGCCCTCAGGTTTGAT-3′
*Cd44*	5′-CTACAGCAAGAAGGGCGAGT-3′	5′-CAAGGTGCTCCGGATAAAGA-3′
*Abcg2*	5′-AGCCTTCCAAGGGAGAGAAG-3′	5′-AGGCCGATGTTCCTTTCTTT-3′
*Abcb1b*	5′-ATGTAGCAAACCTCGGGACA-3′	5′-GGCCAGACAACAGCTTCATT-3′
*Vegf*	5′-CTGCTGTAACGATGAAGCCCTG-3′	5′-GCTGTAGGAAGCTCATCTCTCC-3′

## Data Availability

The datasets generated and/or analysed during the current study are available in the RepOD repository, https://doi.org/10.18150/W0JPSL.

## Supplementary Materials

The following supporting information can be downloaded at https://www.mdpi.com/article/10.3390/cells15151393/s1. Figure S1: Functional effects of *Stat3* silencing on chemotherapy-resistant cells.
