# Supplementary material for: Development of a Doxorubicin Resistance Model in HER2− and HER2+ Breast Cancer to Analyze Potential Therapy Targets and Drug Delivery Methods
Source: Cells. 2026 Jul 31;15(15):1393. doi: 10.3390/cells15151393 (PMC13465200; doi:10.3390/cells15151393)
Supplement: Supplementary file 1 [file cells-15-01393-s001.zip › cells-4424024-supplementary.pdf]

Supplementary

Development of a doxorubicin resistance model in HER2- and HER2+ breast cancer to analyze potential therapy targets and drug delivery methods.

Sara Molenda [1,2,3], Katarzyna Gryska [1, 2], Igor Piotrowski [4], Agata Kubicka[5,6], Agata Sikorska [1, 2], Tomasz Deptuch [1,2], Hanna Dams-Kozlowska [1,2,*]

[1] Department of Cancer Immunology, Poznan University of Medical Sciences, Poland;
[2] Department of Diagnostics and Cancer Immunology, Greater Poland Cancer Centre, Poznan, Poland;
[3] Doctoral School, Poznan University of Medical Sciences, Poland;
[4] Department of Medical Physics, Greater Poland Cancer Centre, Poznan, Poland;
[5] Department of Tumour Pathology and Prophylaxis, Poznan University of Medical Sciences, Poznan, Poland;
[6] Department of Cancer Pathology, Greater Poland Cancer Centre, Poznan, Poland.

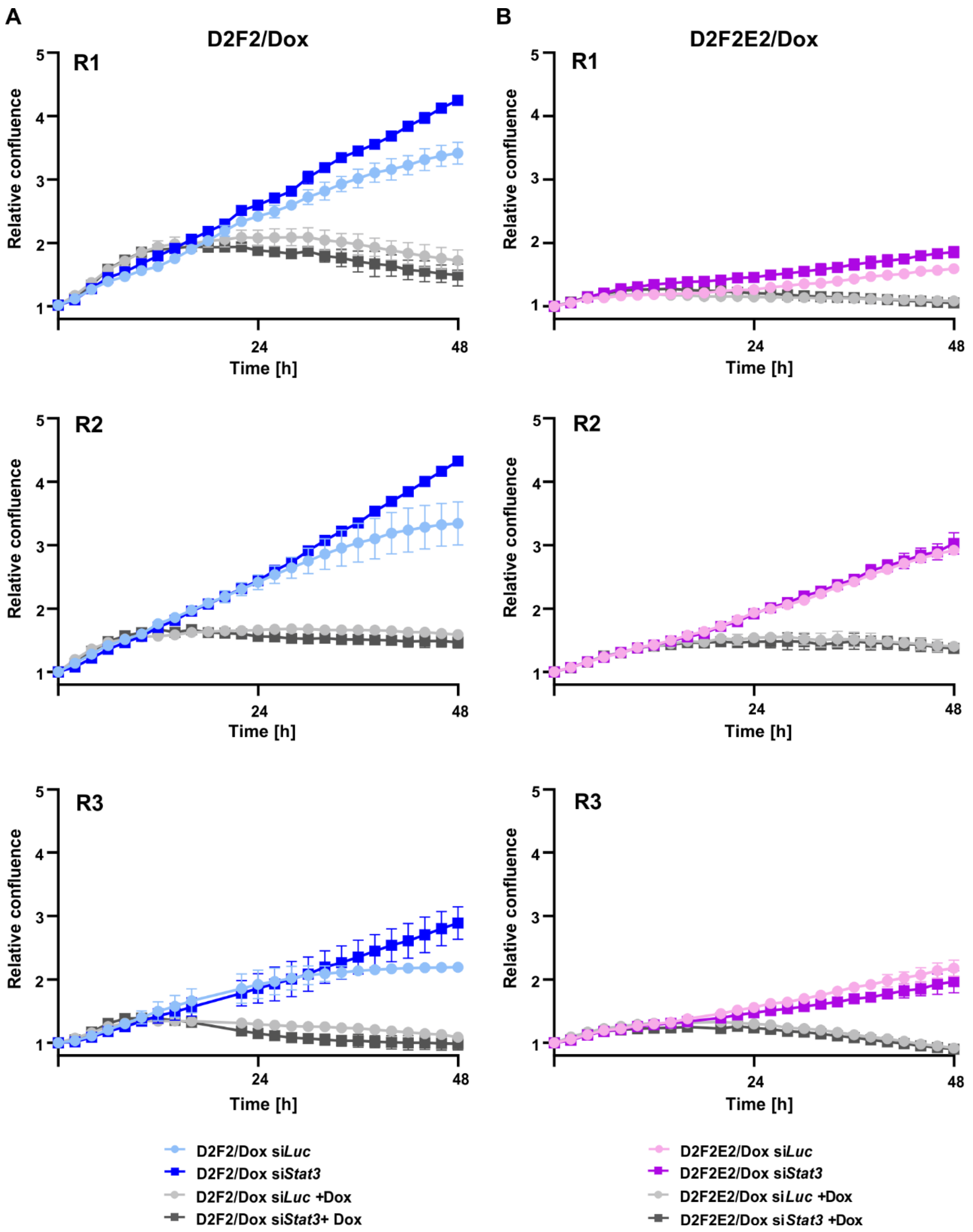

Figure S1. Functional effects of *Stat3* silencing on chemotherapy-resistant cells. D2F2/Dox (A) and D2F2E2/Dox (B) cells were treated with *Stat3*-targeting siRNA (or control *Luc*-targeting siRNA) for 48 hours and subsequently seeded in 96-well plates for further assays: i) cell confluence in the presence of 64 µg/mL doxorubicin was assessed in real-time using IncuCyte ( ), and ii) cell proliferation without presence of chemotherapeutics was monitored in real-time by measuring confluence using the IncuCyte system (and). Each experiment (R1, R2, R3) was performed triplicate (mean ± SEM). Data were analyzed using IncuCyte software and GraphPad Prism.
